# Effect of Environmental and Feedback Interventions on Pacing Profiles in Cycling: A Meta-Analysis

**DOI:** 10.3389/fphys.2016.00591

**Published:** 2016-12-05

**Authors:** Michael J. Davies, Bradley Clark, Marijke Welvaert, Sabrina Skorski, Laura A. Garvican-Lewis, Philo Saunders, Kevin G. Thompson

**Affiliations:** ^1^University of Canberra Research Institute for Sport and ExerciseBruce, ACT, Australia; ^2^Department of Physiology, Australian Institute of SportBruce, ACT, Australia; ^3^Institute of Sports and Preventive Medicine, Saarland UniversitySaarbrücken, Germany; ^4^Mary Mackillop Institute for Health Research, Australian Catholic UniversityMelbourne, VIC, Australia

**Keywords:** pacing, cycling, hypoxia, deception, hyperoxia, heat-stress, pre-cooling, feedback

## Abstract

In search of their optimal performance athletes will alter their pacing strategy according to intrinsic and extrinsic physiological, psychological and environmental factors. However, the effect of some of these variables on pacing and exercise performance remains somewhat unclear. Therefore, the aim of this meta-analysis was to provide an overview as to how manipulation of different extrinsic factors affects pacing strategy and exercise performance. Only self-paced exercise studies that provided control and intervention group(s), reported trial variance for power output, disclosed the type of feedback received or withheld, and where time-trial power output data could be segmented into start, middle and end sections; were included in the meta-analysis. Studies with similar themes were grouped together to determine the mean difference (MD) with 95% confidence intervals (CIs) between control and intervention trials for: *hypoxia, hyperoxia, heat-stress, pre-cooling*, and various forms of *feedback*. A total of 26 studies with cycling as the exercise modality were included in the meta-analysis. Of these, four studies manipulated oxygen availability, eleven manipulated heat-stress, four implemented pre-cooling interventions and seven studies manipulated various forms of feedback. Mean power output (MPO) was significantly reduced in the middle and end sections (*p* < 0.05), but not the start section of hypoxia and heat-stress trials compared to the control trials. In contrast, there was no significant change in trial or section MPO for hyperoxic or pre-cooling conditions compared to the control condition (*p* > 0.05). Negative feedback improved overall trial MPO and MPO in the middle section of trials (*p* < 0.05), while informed feedback improved overall trial MPO (*p* < 0.05). However, positive, neutral and no feedback had no significant effect on overall trial or section MPO (*p* > 0.05). The available data suggests exercise regulation in hypoxia and heat-stress is delayed in the start section of trials, before significant reductions in MPO occur in the middle and end of the trial. Additionally, negative feedback involving performance deception may afford an upward shift in MPO in the middle section of the trial improving overall performance. Finally, performance improvements can be retained when participants are informed of the deception.

## Introduction

The ability to appropriately distribute energy expenditure throughout an exercise task is critical in order to optimize athletic performance (St. Clair Gibson and Noakes, 2004; Abbiss and Laursen, 2008). In the sport science literature this is known as “pacing” or the “pacing strategy” or “pacing profile” and refers to the self-regulation of power (or velocity) during athletic competitions in which athletes are free to vary their exercise intensity (de Morree and Marcora, 2013; Skorski et al., 2015). Research aimed at understanding the underlying mechanisms influencing the selection of pace during exercise has dramatically increased within the last decade. Based on current research, pacing appears to be regulated by complex relationships between the brain and other physiological systems (St. Clair Gibson and Noakes, 2004; Abbiss and Laursen, 2008). Several models have been proposed to explain this phenomena including: the teleoanticipatory theory (Ulmer, 1996; St. Clair Gibson et al., 2006), the central governor model (Noakes et al., 2001), the perception based model (Tucker, 2009), the pacing awareness model (Edwards and Polman, 2013) and the psychobiological model (Marcora, 2010; Pageaux, 2014). Many of these models, however not all, acknowledge that afferent sensory feedback from various physiological systems is received and regulated within the brain and integrated into the pacing strategy as a person responds to ongoing internal stimuli, as well as environmental factors and other external stimuli (Noakes et al., 2001, 2005; St. Clair Gibson and Noakes, 2004). In addition, factors such as knowledge of the task duration or distance remaining (Swart et al., 2009), memory of prior experiences (Mauger et al., 2009), and motivation and mood (de Morree and Marcora, 2013) are also thought to be important factors in the regulation of exercise intensity.

In recent years, a number of studies have shown that physiological, psychological and environmental factors can affect overall performance and pacing (Tucker and Noakes, 2009). These factors include oxygen availability (Amann et al., 2006; Clark et al., 2007; Tucker et al., 2007; Périard and Racinais, 2016), heat-stress (Peiffer and Abbiss, 2011), wind velocity (Teunissen et al., 2013), hydration status (Dugas et al., 2009), carbohydrate (Abbiss et al., 2008) and caffeine ingestion (Wiles et al., 2006), pre-cooling strategies (Duffield et al., 2010), motivation (Corbett et al., 2012), fatigue (Skorski et al., 2015), deception (Stone et al., 2012; Jones et al., 2016b; Shei et al., 2016), pacing feedback (Thompson et al., 2003, 2004) and music (Atkinson et al., 2004). However, on the basis of existing studies it is still difficult to arrive at an overall conclusion as to whether these manipulations have a negative or positive effect on pacing and performance, and indeed which part of the pacing strategy changes (start, middle and end) during trials.

Recently a number of reviews have attempted to explain the influence of deception (Jones et al., 2013; Williams et al., 2014), decision making (Edwards and Polman, 2013; Renfree et al., 2014; Smits et al., 2014; McCormick et al., 2015) and neurophysiological determinants (Roelands et al., 2013) on pacing. However, to date, a meta-analysis investigating the effect of different environmental and extrinsic manipulations on the actual pacing strategy of trained participants during self-paced time-trials is still lacking. This study resolved to estimate the probability that a difference in pacing strategy, due to interventions such as environmental stressors or feedback manipulation, is practically meaningful. Therefore, the aim of this study was to conduct a meta-analysis to provide an overview as to how these types of manipulations affect pacing strategy and exercise performance.

## Methods

A computerized literature search was undertaken, using 21 different key terms (“athletes,” “pacing,” “strategy,” “hypoxia,” “hyperoxia,” “heat,” “precool,” “feedback,” “deception,” environment; “profile,” “self-paced,” “exercise,” “teleoanticipation,” “central,” “peripheral,” “fatigue,” “time-trial,” “performance,” “experience,” and “perceived exertion”) based on the PRIMSA checklist (Liberati et al., 2009; Beller et al., 2013) and the search strategy proposed by Higgins (2011). Combinations of these words were used to systematically search databases, from the following databases: PubMed, SPORTDiscus and MEDLINE (via EBSCO). The literature search began in June 2013 and concluded at 1st September 2016 and was complemented with citation tracking of key primary and review articles.

### Selection criteria

**Figure 1:**
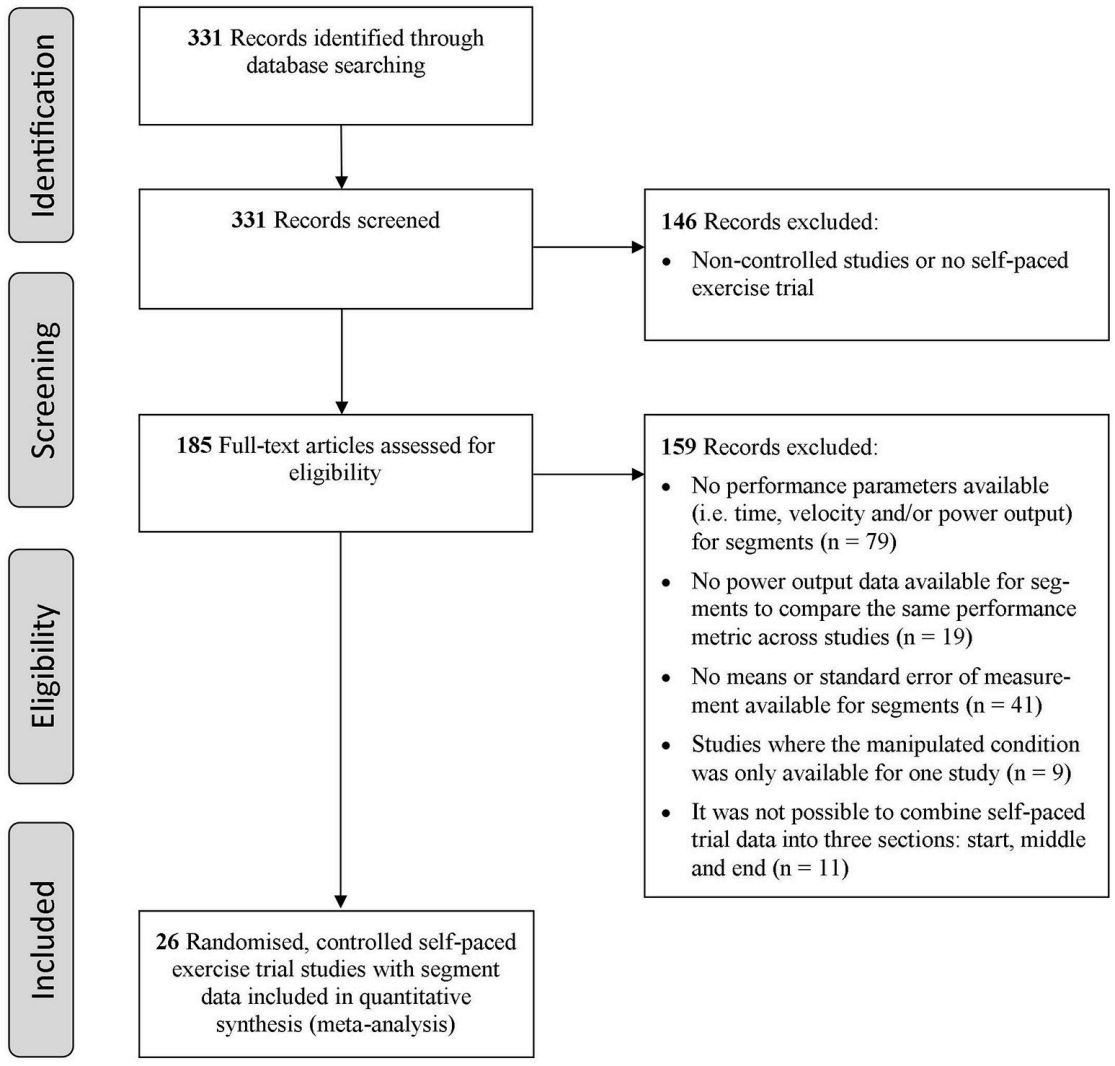
**Flow chart summary of the study selection process**.

Articles were evaluated with respect to their suitability and relevance for the desired context based on the criteria described below. The selection process is also illustrated by a flow chart in Figure 1. Studies not fulfilling these criteria but considered important for the topic are included within the discussion.

A study was only included in the meta-analysis if it fulfilled the following requirements:

1. The existence of a control group or condition without any pacing manipulation, i.e., with the subjects acting as their own controls (randomized crossover design).
2. The performance task had to be reported in terms of at least three sections (start, middle and end) to quantify the effect of an intervention on the pacing strategy of the time-trial. Furthermore, each study had to have measured power output as a performance metric. This parameter was the most commonly reported performance metric in the literature where consistent measurement error data was also provided. Therefore, despite a number of pacing studies providing velocity or time data and meeting all other criteria, they were still excluded as they did not provide power output data. As a consequence, modes of exercise researched in the literature such as running, swimming, skating and rowing were excluded.
3. Pacing data had to be reported as mean and standard deviation (SD), and/or standard error of the mean (SEM), either in tables or figures. Three studies presented their data using SEM (Tatterson et al., 2000; Amann et al., 2006; Byrne et al., 2011), to normalize these data they were converted back to SD values by the primary author (SD = SEM × √*N*).
4. Studies were only included if the trial was self-paced. If this information was not disclosed in the paper, the corresponding author was contacted. When pacing was influenced by an outside source (e.g., coach or researcher) the study was excluded. Studies manipulating the starting strategy or which incorporated intermittent “efforts” during a trial were also excluded.
5. Studies must have indicated whether or not participants received feedback and the type of feedback received (e.g., elapsed time, distance or power output during the trial). If this information was not disclosed in the paper, the corresponding author was contacted.
6. The study must have been published in an internationally peer-reviewed scientific journal.

### Classification of the studies

Of the initial 185 peer-reviewed studies identified, a total of 26 studies satisfying the inclusion criteria were analyzed. These studies were comprised of 44 different trial comparisons. Studies were coded for the descriptive environmental or extrinsic variables manipulated by the researchers. For example, environmental conditions (fraction of inspired oxygen levels (F_*i*_O_2_), temperature, humidity and wind velocity) and feedback received or withheld during the trials. As a result, four different themes (or groups) were identified: (1) oxygen availability (e.g., hypoxia, F_*i*_O_2_ < 0.21; normoxia ~0.21; iso-oxia, F_*i*_O_2_ > 0.21 to 0.30; hyperoxia F_*i*_O_2_ > 0.30), (2) heat-stress, (3) pre-cooling strategies prior to trials in hot conditions (e.g., wearing a cooling vest or cold water immersion), and (4) feedback (e.g., full or no feedback, positive, negative, neutral or informed feedback).

Feedback groups were defined as the following: *full feedback*, where all available feedback was provided and given accurately (control trial); *no feedback*, where all feedback was withheld; *neutral feedback*, where participants raced a virtual on-screen avatar that accurately represented the mean power output (MPO) of a previous performance; *positive deceptive feedback*, where participants were informed they were performing better than in reality (e.g., informed they had traveled a greater distance than they actually had or informed that the ambient and their core temperatures were lower than they actually were); *negative deceptive feedback*, where participants were informed they were performing worse than they were in reality or where performance feedback was inaccurate. For example, performance deception, where participants competed against a previous performance, where the MPO was increased compared to the previous (baseline) trial and participants were either aware (Jones et al., 2016a,b) or unaware (Jones et al., 2016a,b; Shei et al., 2016); and finally *informed feedback*, where participants completed a final trial after being informed that their previous trial in the presence of a pacer was set at a greater exercise intensity than their baseline trial (Jones et al., 2016a; Shei et al., 2016).

### Data extraction

For all studies, power output was extracted for the control and intervention conditions for MPO of the whole trial and each section of the trial (start, middle and end). A large proportion of the analyzed studies (*n* = 25) displayed their results in figures, hence the mean and SD were measured from plots and error bars by the primary researcher using a hand T-square ruler measuring to the nearest millimeter. Each figure was enlarged to A3 size, printed and fixed to a bench. Mean values were measured from the middle of each plot and SD at the top edge of each error bar for every segment. In order to prevent a bias all measurements were repeated exactly a month later by the same person, following the same protocol. Intra-rater reliability between measurements was calculated using the statistical Software R (Vienna, Austria: R Foundation for Statistical Computing). To estimate the level of agreement between the two measures the intra-class correlation coefficient (ICC) was calculated and interpreted according to the thresholds described by (Landis and Koch, 1977). Analysis revealed an almost perfect correlation between the two measures (ICC = 0.99; 95% confidence intervals (CI): 0.99 and 0.99). We were therefore confident there was minimal researcher and measurement bias when extracting the data for the meta-analysis (Landis and Koch, 1977).

### Study quality assessment

Although it was not a requirement for the inclusion criteria, the PEDro scale (Machado et al., 2016) was used to quantify methodological quality of included studies. Briefly, the PEDro scale assesses research against 11 criteria related to study design, from which a score can be assigned to a specific paper from 0 to 11. A score ≥ 7 is considered “high quality,” a score of 5 or 6 is deemed “moderate quality,” and ≤ 4 defined as “poor quality” (Machado et al., 2016).

### Data analysis

The meta-analysis was performed in statistical Software R (Vienna, Austria: R Foundation for Statistical Computing) using the package metaphor (Viechtbauer, 2010). The mean difference (MD) between control and intervention trial for each study was analyzed using a multi-level random effects model including a random effect for start, middle and end sections to account for the dependencies between results from the same studies, represented by 95% confidence internals (CIs). Separate theme analyses for each section were carried out using a random effects model. Heterogeneity was assessed using the Q statistic, described by the *I*^2^ statistic, and publication bias using funnel plots.

For further comparison of pacing strategies we also calculated the pacing index (IP) (Le Meur et al., 2011; Wu et al., 2014) for control and experimental conditions for all studies that reported overall trial MPO. The IP reports the exercise intensity for each segment as a percentage of overall trial MPO and is derived using the following equation:

$$\begin{array}{l} (Segment\;mean\;power\;output/\\ Overall\;trial\;mean\;power\;output)*100 \end{array}$$

As the majority of studies did not report individual participant data it was not possible to calculate a SD for the IP metric and subsequently include in the meta-analysis. Therefore, the IP data were analyzed using an exploratory graphical analysis of the difference between control and experimental condition IP (i.e., control IP—experimental IP for each individual segment).

## Results

### Search results

In total 331 articles were found, out of which 185 were identified as peer-reviewed controlled studies. These articles were evaluated according to the specified inclusion criteria (Figure 1) and we identified 26 studies with a total number of 351 subjects that met all inclusion criteria. Almost all studies used a randomized cross-over design, except for studies (Williams et al., 2012; Jones et al., 2016a,b; Schmit et al., 2016; Smits et al., 2016) which used a parallel group design.

Four studies manipulated oxygen availability, 15 manipulated environmental temperature or implemented a pre-cooling intervention in heat, and seven studies manipulated feedback. In all of these studies cycling was the chosen exercise mode. Specifically, investigations manipulating environmental conditions included: *hypoxia* (*n* = 3), *hyperoxia* (*n* = 2), *heat-stress* (*n* = 11) and *pre-cooling interventions* in the heat (*n* = 4). Seven investigations manipulated feedback however a number of different manipulations were undertaken including: *positive deceptive feedback* (*n* = 3), *negative deceptive feedback* (e.g., performance deception, *n* = 3), *neutral feedback* (*n* = 2), *no feedback* (*n* = 3) and *informed feedback* (*n* = 2). One study (Castle et al., 2012) analyzed the effects of heat-stress or deceiving participants of ambient and core temperature by providing positive deceptive feedback, thus data were included in two themes, heat-stress and feedback, respectively. Participants' maximal oxygen uptake ($\dot{V}$O_2max_) from the 19 studies which reported this parameter was (mean ± SD) 61.3 ± 4.6 mL^−1^.kg^−1^.min. Three studies only provided peak power output (PPO) data (393 ± 51 W), while four studies did not disclose participants' $\dot{V}$ O_2*max*_ or PPO. Most studies exclusively investigated male participants, except for one, which reported having one female participant amongst a male cohort (Périard and Racinais, 2015). However, one study did not report the gender of their participants (Schmit et al., 2016). An overview of included studies is provided in Table 1.

**Table 1:** **Overview of the analyzed studies**.

**Study, year**	**Study design**	**No. of participants and training status^a^**	**Exercise protocol**	**Intervention**	**Time point of environmental exposure or intervention manipulation**	**Performance measurement (total time or mean power output (MPO) in watts)**	**PEDro Score**
**OXYGEN AVAILABILITY**							
Amann et al., 2006	Crossover	8 Well-trained male cyclists (63.3 ± 1.3 ml kg^−1^min^−1^)	5 km cycling time-trial	Time-trial in normoxia (FiO_2_ = 0.21; CON.), hypoxia (FiO_2_ = 0.15; INTER), iso-oxia (FiO_2_ = 0.24–0.30; INTER) and hyperoxia (FiO_2_ = 1.00; INTER)	Exposure 3 min before trial, after a warm-up in normoxia	FiO_2_: 0.21 = 458 ± 7 s FiO_2_: 0.15 = 483 ± 8 s FiO_2_: 0.24–0.30 = 351 ± 7 s FiO_2_: 1.00 = 439 ± 7 s	9
Clark et al., 2007	Crossover	10 Well-trained male cyclists and triathletes (67.7 ± 1.3 ml kg^−1^min^−1^)	5 min cycling time-trial	Time-trial in normoxia (FiO_2_ = 0.21; CON) and hypoxia (FiO_2_ = 0.19; 0.16; and 0.14 INTER)	Exposure before, during and after warm-up. Total of 40 min in hypoxia before trial	FiO_2_: 0.21 = 367 ± 42 MPO FiO_2_: 0.19 = 346 ± 41 MPO FiO_2_: 0.16 = 329 ± 38 MPO FiO_2_: 0.14 = 294 ± 37 MPO	10
Périard and Racinais, 2016	Crossover	12 Well-trained male cyclists	Cycling time-trial to complete 750 kJ	Time-trial in normoxia (FiO_2_ = 0.21; CON) and hypoxia (FiO_2_ = 0.15; INTER)	Exposure 5 min before trial, after a warm-up in normoxia	FiO_2_: 0.21 = 48.2 ± 5.7 min FiO_2_: 0.15 = 60.1 ± 6.5 min	9
Tucker et al., 2007	Crossover	11 Well-trained male cyclists (395 ± 33 PPO)	20 km cycling time-trial	Time-trial in normoxia (FiO_2_ = 0.21; CON) and hyperoxia (FiO_2_ = 0.40; INTER)	Time point unknown, including oxygen inhaled in warm-up	FiO_2_: 0.21 = 28 ± 8 min FiO_2_: 0.40 = 27 ± 34 min	9
**HEAT-STRESS**							
Abbiss et al., 2008	Crossover	10 Well-trained male cyclists (61.7 ± 5.0 ml kg^−1^min^−1^)	16.1 km cycling time-trial	Time-trial in temperate (18.1°C, 58% RH; CON) and heat (32°C, 50% RH; INTER)	Exposure during warm-up (90 min fixed workload) and at rest. Total of 116.3 min in heat before trial	18.1°C = 25.4 ± 1.6 min 32°C = 27.5 ± 1.9 min	8
Altareki et al., 2009	Crossover	9 Competitive male triathletes and cyclists (61.7 ± 8.6 ml kg^−1^min^−1^)	4 km cycling time-trial	Time-trial in cool (13°C, 40%; CON) and heat (35°C, 60% RH; INTER)	Exposure before, during and after warm-up. Total of 21 min in heat before trial	13°C = 382.8 ± 18.2 s 35°C = 390.1 ± 19.6 s	8
Castle et al., 2012	Crossover	7 Recreational male cyclists (58.8 ± 5.7 ml kg^−1^min^−1^)	30 min cycling time-trial	Time-trial in temperate (22°C, 43% RH; CON) and heat (31°C, 64% RH; INTER)	Exposure during a 7 min warm-up in heat before trial	22°C = 179.9 ± 50.9 MPO 31°C = 168.1 ± 54.1 MPO	9
Peiffer and Abbiss, 2011	Crossover	9 Trained male cyclists (60.5 ± 4.5 ml kg^−1^min^−1^)	40 km cycling time-trial	Time-trial in temperate (22°C, 40% RH; CON), cool (17°C, 40% RH; INTER), warm (27°C 40% RH; INTER), and hot (32°C, 40% RH; INTER)	Time point unknown, including temperature in warm-up	17°C = 58.8 ± 2.0 min 22°C = 59.0 ± 2.3 min 27°C = 59.1 ± 2.3 min 32°C = 60.7 ± 2.9 min	8
Périard et al., 2011	Crossover	8 Well-trained male cyclists (66.4 ± 5.3 ml kg^−1^min^−1^)	40 km cycling time-trial	Time-trial in temperate (20°C, 40%: CON) and heat (35°C, 60% RH; INTER)	Exposure 5 min before trial, after a warm-up in temperate	20°C = 59.8 ± 2.6 min 35°C = 64.3 ± 2.8 min	8
Périard and Racinais, 2015	Crossover	11 Well-trained cyclists, 10 males and 1 female (60.2 ± 6.3 ml kg^−1^min^−1^)	Cycling time-trial to 750 kJ complete	Time-trial in temperate (20°C, 40%; CON) and heat (35°C, 60% RH; INTER)	Exposure 5 min before trial, after a warm-up in temperate	20°C = 48.8 ± 12.7 min 35°C = 55.8 ± 14.4 min	8
Périard and Racinais, 2016	Crossover	12 Well-trained male cyclists	Cycling time-trial to 750 kJ complete	Time-trial in temperate (18°C, 40%; CON) and heat (35°C, 60% RH; INTER)	Exposure 5 min before trial, after a warm-up in temperate	18°C = 48.2 ± 5.7 min 35°C = 55.4 ± 5.0 min	8
Schmit et al., 2016	Parallel group	34 Well-trained triathletes, gender unknown. Temperate (*n* = 22; 63.3 ± 2.1 ml kg^−1^min^−1^), Heat (*n* = 12; 62.2 ± 3.6)	20 km cycling time-trial	Time-trial in temperate (21°C, 50%; CON) acute heat exposure (35°C, 50% RH at 0; INTER and 11 ± 4 days; INTER)	Exposure 15 min before trial during a warm-up, the second heat trail took place 11 ± 4 days after trial 1 with no acclimatization	*0 days* 21°C = 32.2 ± 2.0 min 35°C = 33.2 ± 1.58 min *11* ± *4 days after first trial* 21°C = 31.5 ± 1.4 min 35°C = 32.4 ± 1.2 min	7
Tatterson et al., 2000	Crossover	11 National male road cyclists (66.7 ± 13.6)	30 min cycling time-trial	Time-trial in temperate (23°C, 60% RH; CON) and heat (32°C, 60% RH; INTER)	Time point unknown, including temperature in warm-up	23°C = 345 ± 9 MPO 32°C = 323 ± 8 MPO	8
Tucker et al., 2004	Crossover	10 Recreational male cyclists (376 ± 47 PPO)	20 km cycling time-trial	Time-trial in cool (15°C, 60% RH; CON) and heat (35°C, 60% RH; INTER)	Time point unknown, including temperature in warm-up	15°C = 28.8 ± 1.8 min 35°C = 29.6 ± 1.9 min	8
VanHaitsma et al., 2016	Crossover	20 Trained male cyclists (54.8 ± 5.9)	40 km cycling time-trial	Time-trial in temperate (21°C, 20% RH; CON) and heat (35°C, 25% RH; INTER)	Time point unknown, including temperature in warm-up	21°C = 75.2 ± 6.6 min 35°C = 79.0 ± 7.2 min	8
**PRE-COOLING STRATEGIES**							
Barwood et al., 2012	Crossover	11 Trained male cyclists	40 km cycling time-trial	Time-trial in heat (32°C, 53% RH) with use of no spray (CON), menthol spray (INTER) and placebo cooling spray (INTER)	10 min warm-up in heat, existed chamber (5 min rest in ~22°C), re-entered and sprayed with solution. Total of ~23 min in heat	CON = 71.58 ± 62 min Placebo = 70.94 ± 6.1 min Menthol = 71.04 ± 5.5 min	9
Byrne et al., 2011	Crossover	7 Recreational male cyclists	30 min cycling time-trial	Time-trial in the heat (32°C, 60% RH) ingesting 900mL of hot (37°C; CON) or cold fluid (2°C; INTER) 10 min before trial	After fluid ingestion, 5 min at rest in heat, temperature of warm-up unknown	37°C fluid = 261 ± 22 MPO 2°C fluid = 275 ± 27 MPO	8
Duffield et al., 2010	Crossover	8 Moderate to well-trained male cyclists (lactate threshold 221 ± 42 W)	40 min cycling time-trial	Time-trial in the heat (33°C, 50% RH) with (INTER) and without (CON) lower body cold water immersion (CWI) at 14°C before trial	20 min of CWI, 5 min warm-up in heat. Time between CWI and warm up ~8–10 min, ≤ 5 min from warm-up and trial start	CON = 178 ± 26 MPO 14°C CWI = 198 ± 25 MPO	8
Gonzales et al., 2014	Crossover	10 trained male cyclists (59.1 ± 7.0 ml kg^−1^min^−1^)	20 min cycling time-trial	Time-trial in the heat (30°C, 79% RH) with (INTER) and without (CON) a cooling vest worn and refrigerated headband	18 min warm-up with cooling vest and headband in heat. Rested in heat for 10 min (no vest) before trial. Total of 28 min in heat	CON = 222 ± 47 MPO Cooling vest = 239 ± 45 MPO	8
**FEEDBACK MANIPULATIONS**							
Albertus et al., 2005	Crossover	15 Competitive male cyclists (397 ± 58 PPO)	20 km cycling time-trial	Time-trial with correct fb (CON) and positive deception (Pos-fb; INTER)	Informed traveled 25-m further than they had every km	CON = 28.4 ± 1.6 min Pos-fb = 28.6 ± 1.5 min	8
Castle et al., 2012	Crossover	7 Recreational male cyclists (58.8 ± 5.7 ml kg^−1^min^−1^)	30 min cycling time-trial	Time-trial in the heat without deception (31°C, 64% RH; CON) and with deception (told the temperature was 26°C, 60% RH, reality: 32°C, 65% RH; Pos-fb; INTER)	From the onset of the trial and displayed incorrectly during trial on a computer screen	CON = 179.9 ± 50.9 MPO Pos-fb = 184.4 ± 60.4 MPO	9
Jones et al., 2016b	Parallel group	20 Trained male cyclists, CON (*n* = 10; 57.6 ± 6.7 ml kg^−1^min^−1^), Deception (*n* = 10; 58.7 ± 6.6 ml kg^−1^min^−1^)	16.1 km cycling time-trial	Time-trial with accurate ride-alone fb (CON), against a pacer representing MPO of CON (Neutr-fb; INTER), unaware of MPO of pacer 102% above baseline (Neg-fb; INTER) and a subsequent ride-alone trial (not informed of deception in previous trial and not included in meta-analysis)	From the onset of the trial	*Control Group:* CON = 27:10 ± 2:08 min Neutr-fb = 26:47 ± 1:55 min Inform-fb = 26:55 ± 1:58 min *Deception Group:* CON = 27:00 ± 1:31 min Pos-fb = 26:41 ± 1:13 min Inform-fb = 26:56 ± 1:38 min	9
Jones et al., 2016a	Parallel group	17 Trained male cyclists, CON (*n* = 9; 54.1 ± 5.9 ml kg^−1^min^−1^), Deception (*n* = 8; 53.3 ± 4.4 ml kg^−1^min^−1^)	16.1 km cycling time-trial	Time-trial with accurate ride-alone fb (CON), against a pacer representing MPO of CON (Neutr-fb; INTER), unaware or aware that MPO of pacer was increased 102% above (Neg-fb; INTER) and a subsequent ride-alone trial after being informed of the nature of a previous trial (Inform-fb; INTER)	From the onset of the trial	*Control Group:* CON = 26:31 ± 1:44 min Neutr-fb = 26:15 ± 1:31 min Inform-fb = 26:40 ± 1:30 min *Deception Group:* CON = 26:40 ± 0:52 min Pos-fb = 26:22 ± 0:44 min Inform-fb = 26:34 ± 0:54 min	9
Shei et al., 2016	Crossover	14 Competitive male cyclists (61.6 ± 0.6 ml kg^−1^min^−1^)	4 km cycling time-trial	Time-trial with correct fb ride-alone (CON), unaware of MPO of pacer 102% above baseline (Neg-fb; INTER) and a subsequent trial after deception was revealed with known pacer at 102% (Inform-fb; INTER)	From the onset of the trial	CON = 366.4 ± 3.6 MPO Neg-fb = 358.6 ± 2.7 MPO Inform-fb = 358.1 ± 2.8 MPO	8
Smits et al., 2016	Parallel group	20 Trained male cyclists and triathletes, CON (*n* = 10;53.7 ± 7.1 ml kg^−1^min^−1^) and No-fb (*n* = 10; 59.0 ± 7.7 ml kg^−1^min^−1^)	20 km cycling time-trial	Time-trial with distance only fb (CON) and no fb (No-fb; INTER)	From the onset of the trial, cyclists stopped at completed distance	CON = 28.7 ± 3.7 min No-fb = 31.0 ± 2.8 min	8
Swart et al., 2009	Crossover	12 Competitive cyclists (56.6 ± 6.6 ml kg^−1^min^−1^)	40 km cycling time-trial	Time-trial with distance only fb (CON) and no fb (No-fb; INTER)	From the onset of the trial, at final km, cyclists were then informed they had 1-km to complete	CON = 265.5 ± 36.4 MPO No-fb = 256.6 ± 36.6 MPO	8
Waldron et al., 2015	Crossover	9 Well-trained male cyclists (60.5 ± 3.3 ml kg^−1^min^−1^)	4 km cycling time-trial	Time-trial with correct ascending fb (CON) and increased ascending fb by 102% compared to CON (Pos-fb; INTER)	An ascending distance clock was continuously displayed and visible to participants	CON = 354 ± 39 s Pos-fb = 372 ± 36 s	9
Williams et al., 2012	Parallel group	22 Non-competitive and untrained males in cycling, CON (*n* = 11) and No-fb (*n* = 11) groups (50 ± 9 ml kg^−1^min^−1^)	4 km cycling time-trial	Time-trial with distance only fb (CON) and no fb (No-fb; INTER)	From the onset of the trial, cyclists stopped at completed distance	*Group 1* CON = 198 ± 39 MPO No-fb = 179 ± 47 MPO *Group 2* CON = 181 ± 37 MPO No-fb = 171 ± 53 MPO	9

a *Data for VO_2max_ in mL/kg/min and peak power output in watts are presented in mean ± standard deviations. PPO, peak power output; FiO_2_, fraction of inspired oxygen; RH, relative humidity; CON, control trial; INTER, intervention trial; fb, feedback; Pos-fb, positive deceptive feedback; Neg-fb, negative deceptive feedback; Neutr-fb, neutral feedback; No-fb, no; Inform-fb, informed of the nature of a previous trial*.

**Figure 2:**
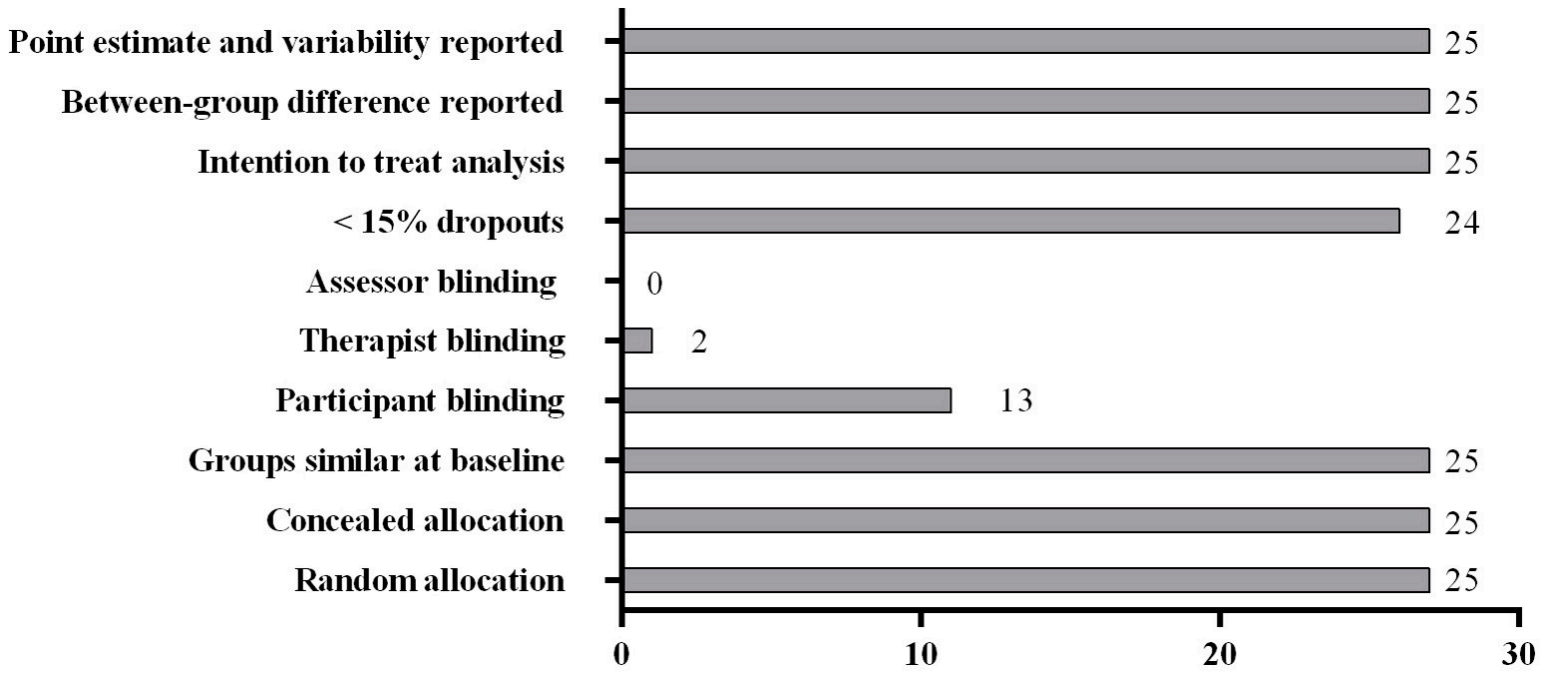
**Number of studies meeting individual PEDro [Physiotherapy Evidence Database] criteria**.

Finally, when studies were assessed using the PEDro scale, all 26 studies were considered as “high quality,” with a PEDro score of (mean ± SD) 8.4 ± 0.6. Due to the nature of heat intervention trials, the blinding of participants is not possible, however in other conditions it is possible to blind participants to the intervention condition and in eleven studies this was the case. In these studies participants were blinded to a manipulation in: oxygen availability (*n* = 4) (Amann et al., 2006; Clark et al., 2007; Tucker et al., 2007; Périard and Racinais, 2016), pre-cooling (*n* = 1) (Barwood et al., 2012) and feedback (*n* = 6) (Albertus et al., 2005; Castle et al., 2012; Waldron et al., 2015; Jones et al., 2016a,b; Shei et al., 2016). Only one study implemented a double-blind design (Clark et al., 2007). Figure 2 illustrates the number of research trials that fulfilled each criteria of the PEDro scale.

### Analysis of studies which investigated the manipulation of hypoxia

**Figure 3:**
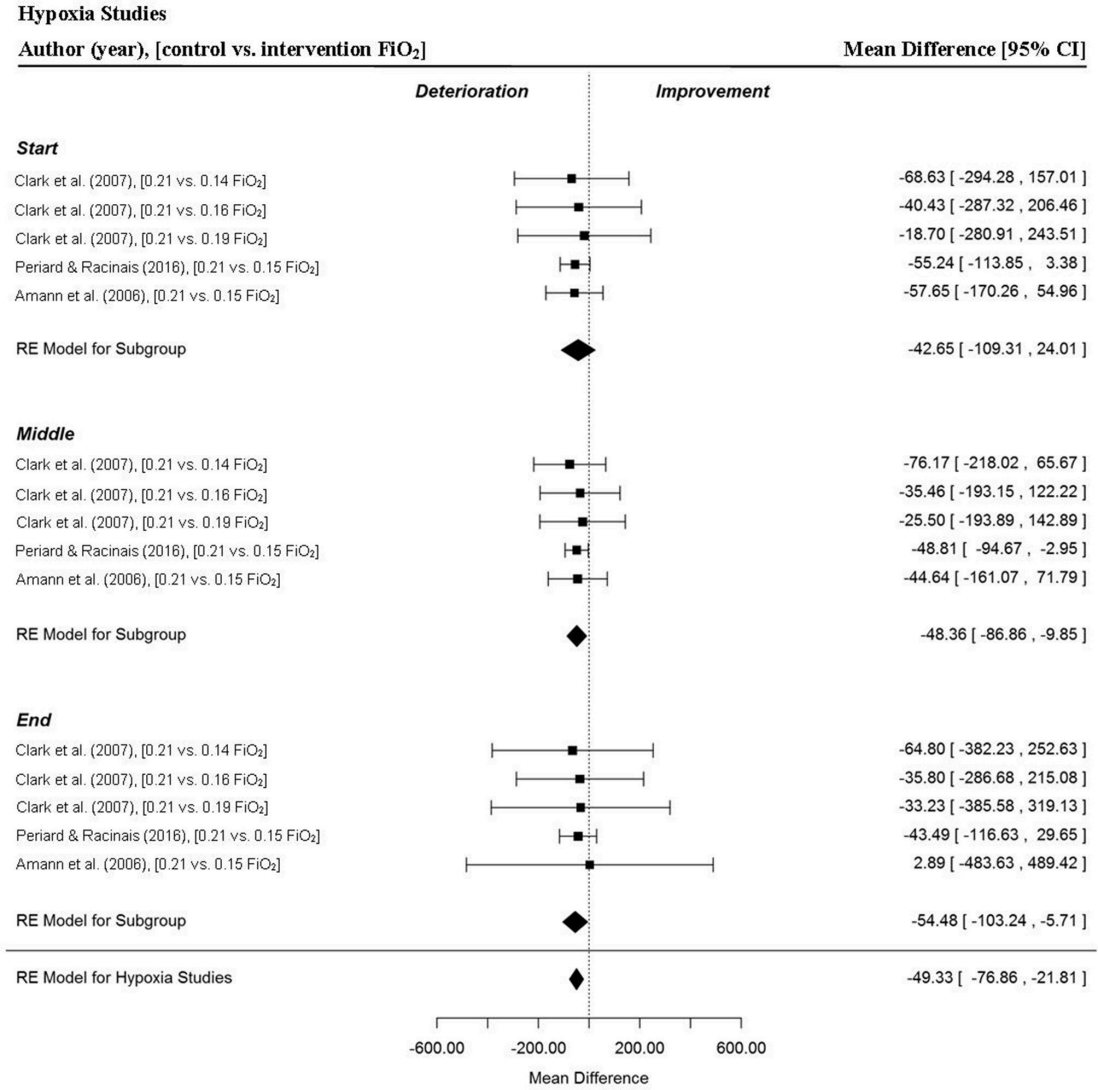
**Forest plot for hypoxia meta-analysis illustrating power output during start, middle and end sections compared to normoxia trials**. Squares represent individual study mean difference and the lines represent 95% CIs. The size of the square is proportional to the weight of the study within the meta-analysis. The diamond represents the overall mean difference for each split with the width of the diamond signifying the 95% CIs.

A total of five MPO data points were extracted from three studies for the start, middle and end sections of trials and overall MPO (Amann et al., 2006; Clark et al., 2007; Périard and Racinais, 2016). Mean power output was significantly reduced for all intervention trials (Mean Difference (MD) = −49.33 watts (W), 95% confidence interval (95% CI) = −76.89 to −21.81, *p* = 0.000) compared to the control (normoxia) trial (Figure 3). There was a significant reduction in MPO for the middle (MD = −48.36 W, 95% CI = −86.86 to −9.95, *p* = 0.014) and end sections (MD = −54.48 W, 95% CI = −103.24 to −5.71, *p* = 0.029) of the intervention trials. Interestingly, one study reported no meaningful changes across all trial sections for low (0.19 F_i_O_2_), moderate (0.16 F_i_O_2_) and high (0.14 F_i_O_2_) stimulated altitude compared to sea level (Clark et al., 2007). In addition, a large variation in section MPO, indicated by the range of the 95% CIs, was observed across all three sections of the intervention trials compared to the control (normoxia) trial in this study (Figure 3). One study (Amann et al., 2006) demonstrated a marked reduction in the pacing index change score (**Figure 12A**) across the trial for the hypoxic trial IP (FiO_2_ 0.15) relative to the control (normoxic) trial condition.

### Analysis of studies which investigated the manipulation of hyperoxia

**Figure 4:**
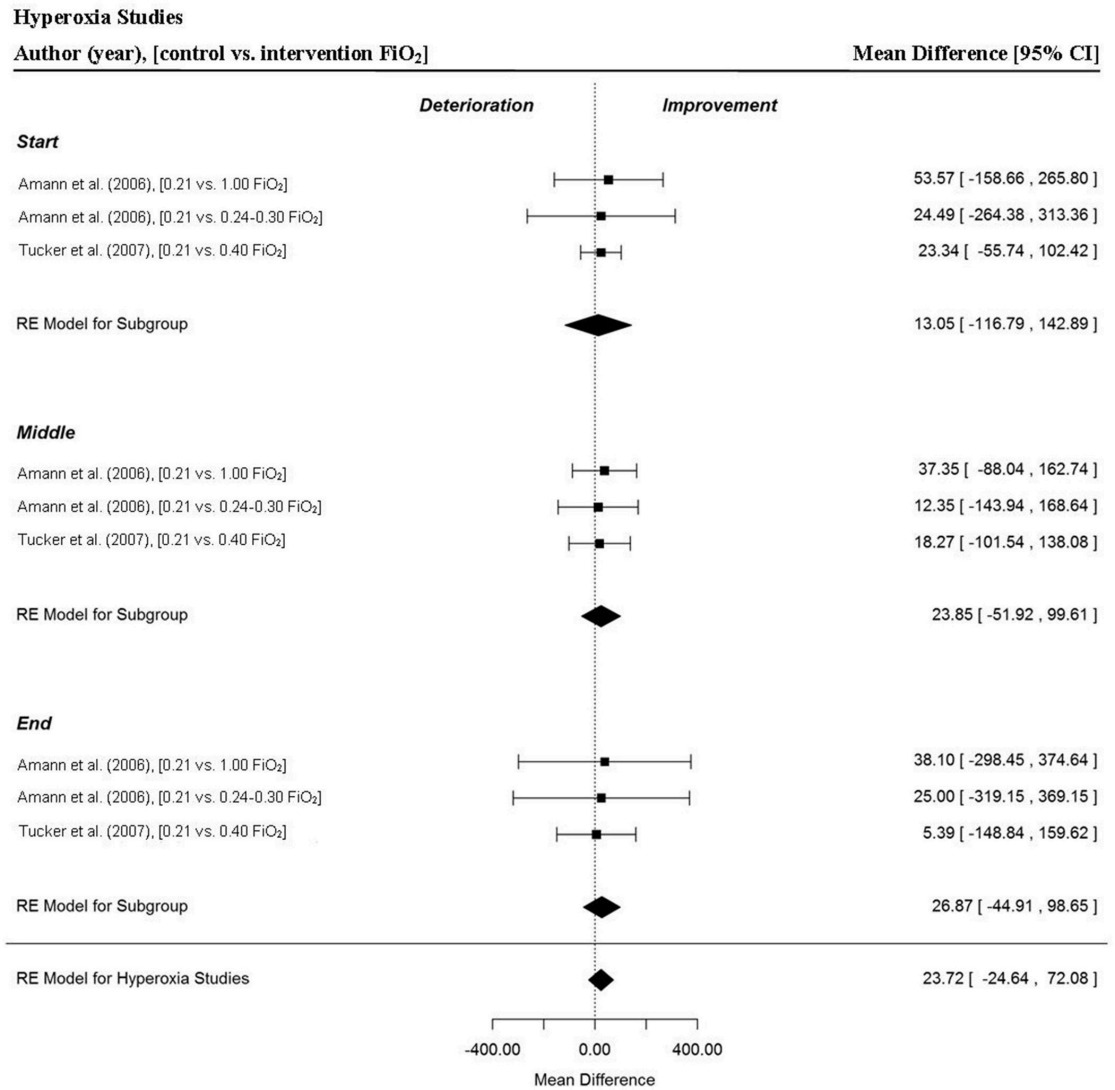
**Forest plot for hyperoxia meta-analysis illustrating power output during start, middle and end sections compared to normoxia trials**. Squares represent individual study mean difference and the lines represent 95% CIs. The size of the square is proportional to the weight of the study within the meta-analysis. The diamond represents the overall mean difference for each split with the width of the diamond signifying the 95% CIs.

Two studies provided a total of three MPO data points for the start, middle and end sections and for overall time-trials (Amann et al., 2006; Tucker et al., 2007). There was no significant change in overall MPO for trials completed in hyperoxia (MD = 23.72 W, 95% CI = −24.64 to 72.08, *p* = 0.336). Furthermore, power output remained relatively stable through the start (MD = 13.05 W, 95% CI = −116.79 to 142.89, *p* = 0.844), middle (MD = 23.85 W, 95% CI = −51.92 to 99.61, *p* = 0.537) and end sections (MD = 26.87 W, 95% CI = −44.91 to 98.65, *p* = 0.463) of the hyperoxia trials respectively (Figure 4).

### Analysis of studies which investigated the manipulation of heat-stress

**Figure 5:**
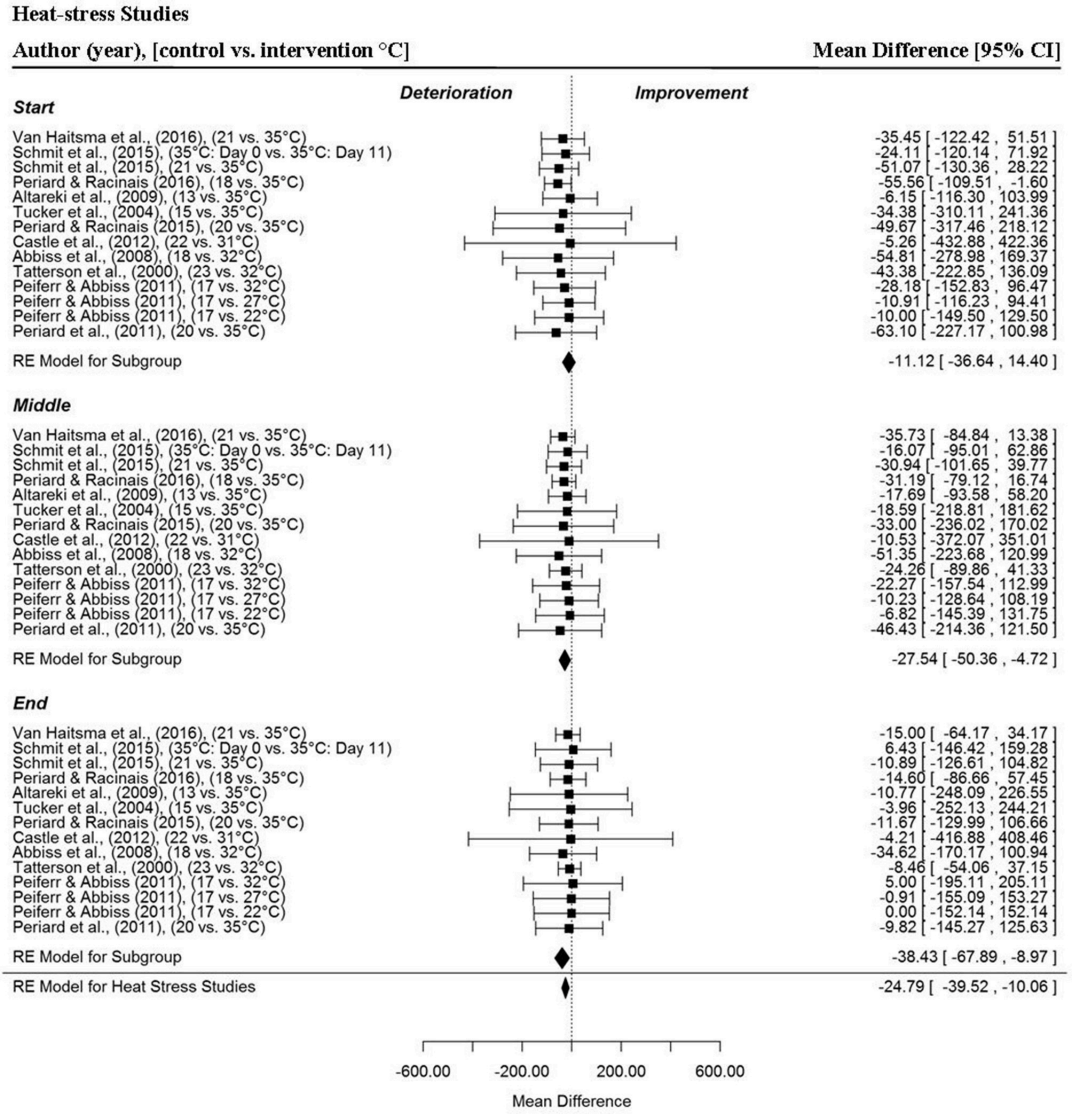
**Forest plot for heat-stress meta-analysis illustrating power output during start, middle and end sections compared to temperate trials**. Squares represent individual study mean difference and the lines represent 95% CIs. The size of the square is proportional to the weight of the study within the meta-analysis. The diamond represents the overall mean difference for each split with the width of the diamond signifying the 95% CIs.

Heat-stress (Figure 5) had a significant, negative impact on time-trial performance from a sample of 14 MPO data points per trial section MPO and for the whole trial MPO across 11 included studies (MD = −24.79 W, 95% CI = −39.52 to −10.06, *p* = 0.001) (Tatterson et al., 2000; Tucker et al., 2004; Abbiss et al., 2008; Altareki et al., 2009; Peiffer and Abbiss, 2011; Périard et al., 2011; Castle et al., 2012; Périard and Racinais, 2015, 2016; Schmit et al., 2016; VanHaitsma et al., 2016). Trial section data demonstrated a gradual decline in MPO (and Pacing Index Change Score) as the trials progressed under hot conditions compared to the control condition (**Figure 12**). Mean power output was significantly reduced in the middle (MD = −27.54 W, 95% CI = −50.36 to −4.72, *p* = 0.018) and end sections (MD = −38.43 W, 95% CI = −67.89 to −8.97, *p* = 0.011) but not the start section (MD = −11.12 W, 95% CI = −36.64 to 14.40, *p* = 0.393).

### Analysis of studies which investigated the manipulation of pre-cooling strategies

**Figure 6:**
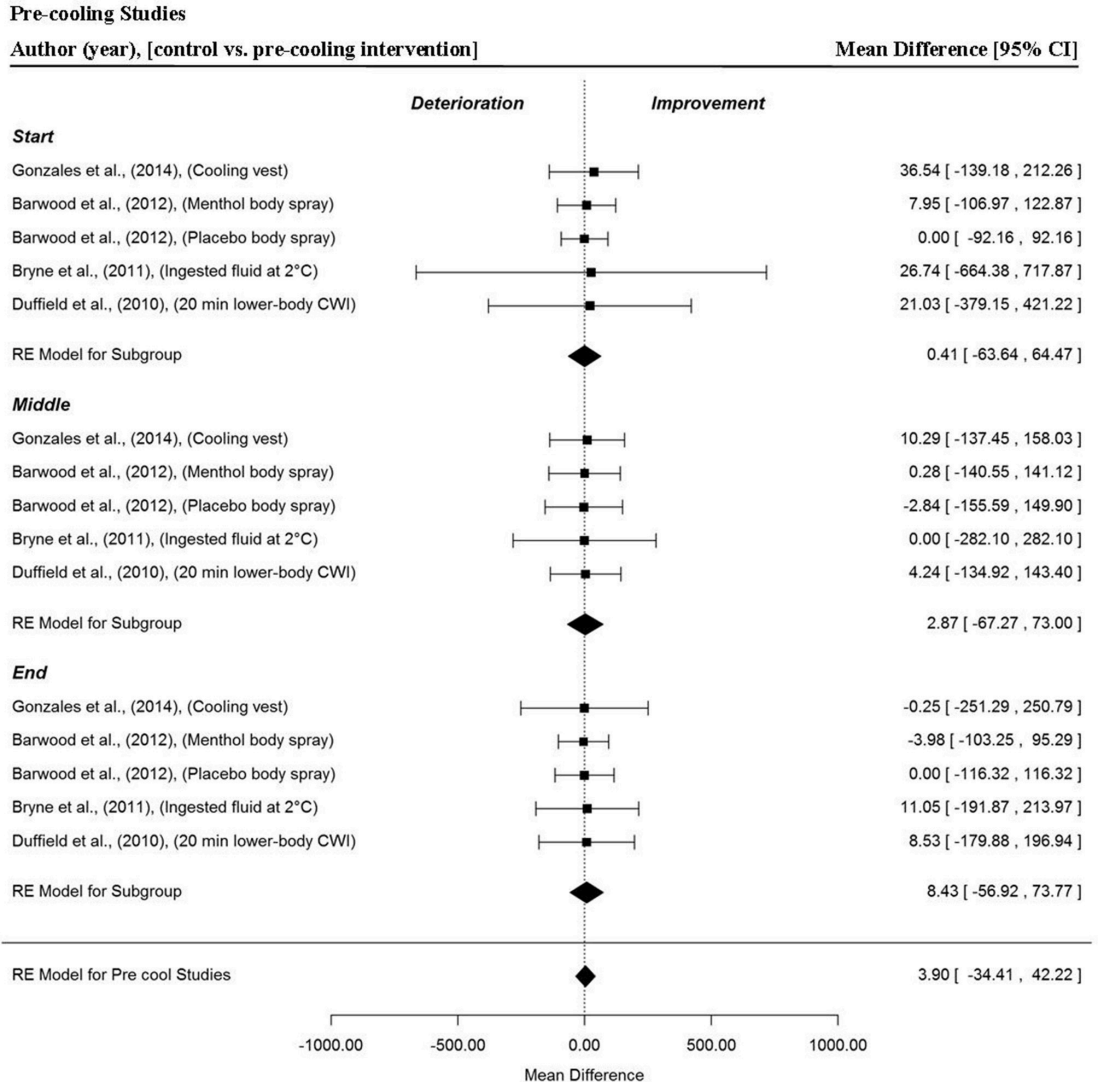
**Forest plot for pre-cooling intervention meta-analysis illustrating power output during start, middle and end sections power output for start, middle and end sections compared to no intervention**. Squares represent individual study mean difference and the lines represent 95% CIs. The size of the square is proportional to the weight of the study within the meta-analysis. The diamond represents the overall mean difference for each split with the width of the diamond signifying the 95% CIs.

Five data points per trial section MPO and for the whole trial MPO were extracted from four studies (Duffield et al., 2010; Byrne et al., 2011; Barwood et al., 2012; Gonzales et al., 2014), to analyse the effect of pre-cooling interventions in hot conditions on time-trial performance. Overall, no significant difference in trial MPO was detected in pre-cooling trials compared to control (no cooling intervention) (MD = 3.90 W, 95% CI = −34.41 to 42.22, *p* = 0.842). The meta-analysis detected no significant changes in MPO for the start (MD = 0.41 W, 95% CI = −63.64 to 64.47, *p* = 0.990), middle (MD = 2.87 W, 95% CI = −67.27 to 73.00, *p* = 0.934) and end (MD = 8.43 W, 95% CI = −56.92 to 73.77, *p* = 0.801) sections in the four studies (Figure 6).

### Analysis of studies which investigated the manipulation of positive deceptive feedback

**Figure 7:**
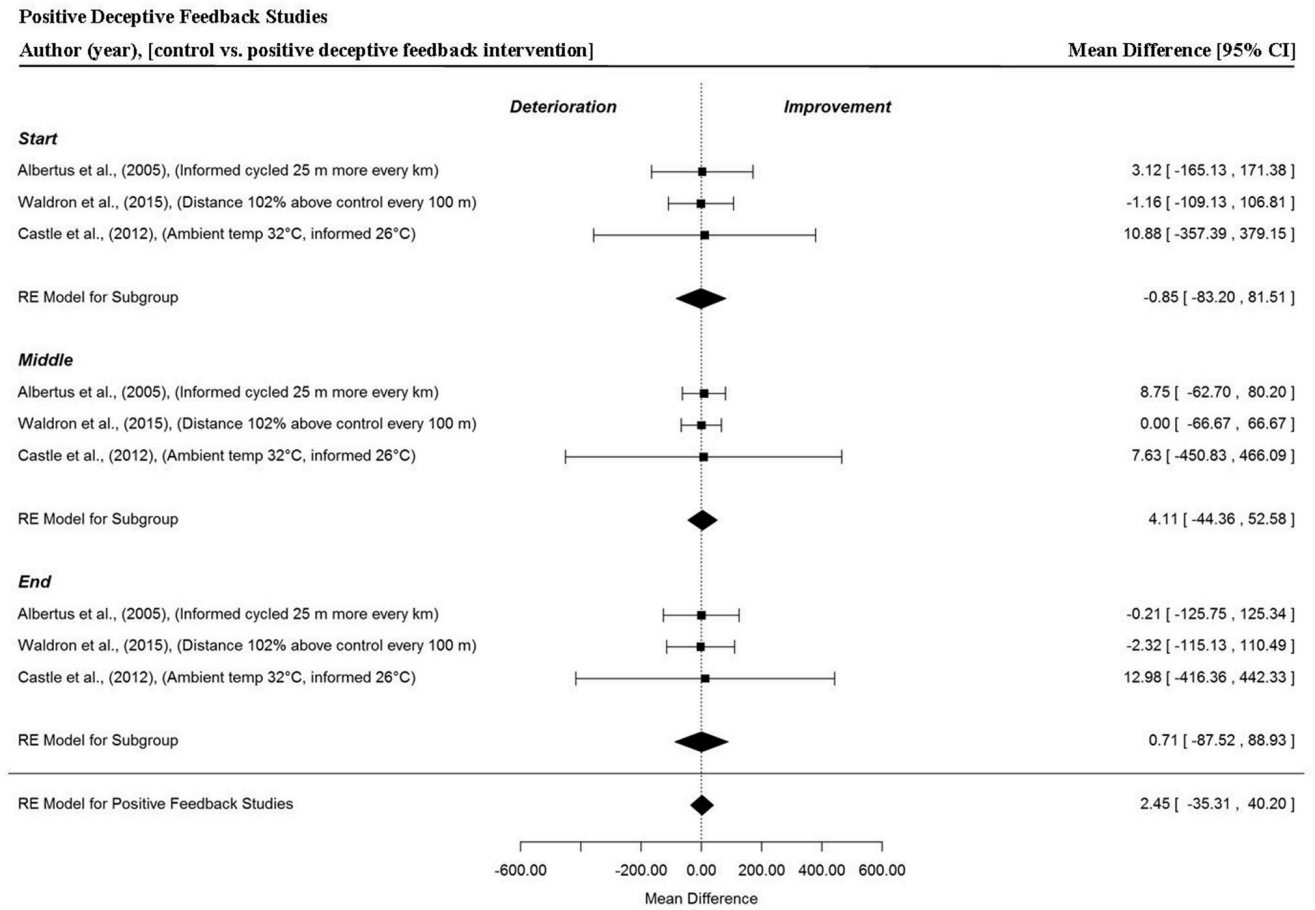
**Forest plot for positive deceptive feedback meta-analysis illustrating power output during start, middle and end sections power output for start, middle and end sections compared to full feedback**. Squares represent individual study mean difference and the lines represent 95% CIs. The size of the square is proportional to the weight of the study within the meta-analysis. The diamond represents the overall mean difference for each split with the width of the diamond signifying the 95% CIs.

Overall and trial section MPO was extracted from three studies that provided three data points per section (Albertus et al., 2005; Castle et al., 2012; Waldron et al., 2015). There was no significant change in trial MPO when cyclists were provided with positive deceptive feedback (MD = 2.45 W, 95% CI = −35.31 to 40.20, *p* = 0.899). Furthermore, this trend was apparent across the start (MD = −0.85 W, 95% CI = −83.20 to −81.51, *p* = 0.984), middle (MD = 4.11 W, 95% CI = −44.36 to 52.58, *p* = 0.868) and end (MD = 0.71 W, 95% CI = −87.52 to 88.93, *p* = 0.987) sections of trials. Notably, wider 95% CIs were found when cyclists received positive deceptive feedback compared to the other feedback groups, indicating a greater variability in overall performance and section MPO with this form of feedback (Figure 7). This was particularly evident, when cyclists were informed that their core and environmental temperature were lower (0.3° and 26°C, respectively) compared to control (Castle et al., 2012).

### Analysis of studies which investigated the manipulation of negative deceptive feedback

**Figure 8:**
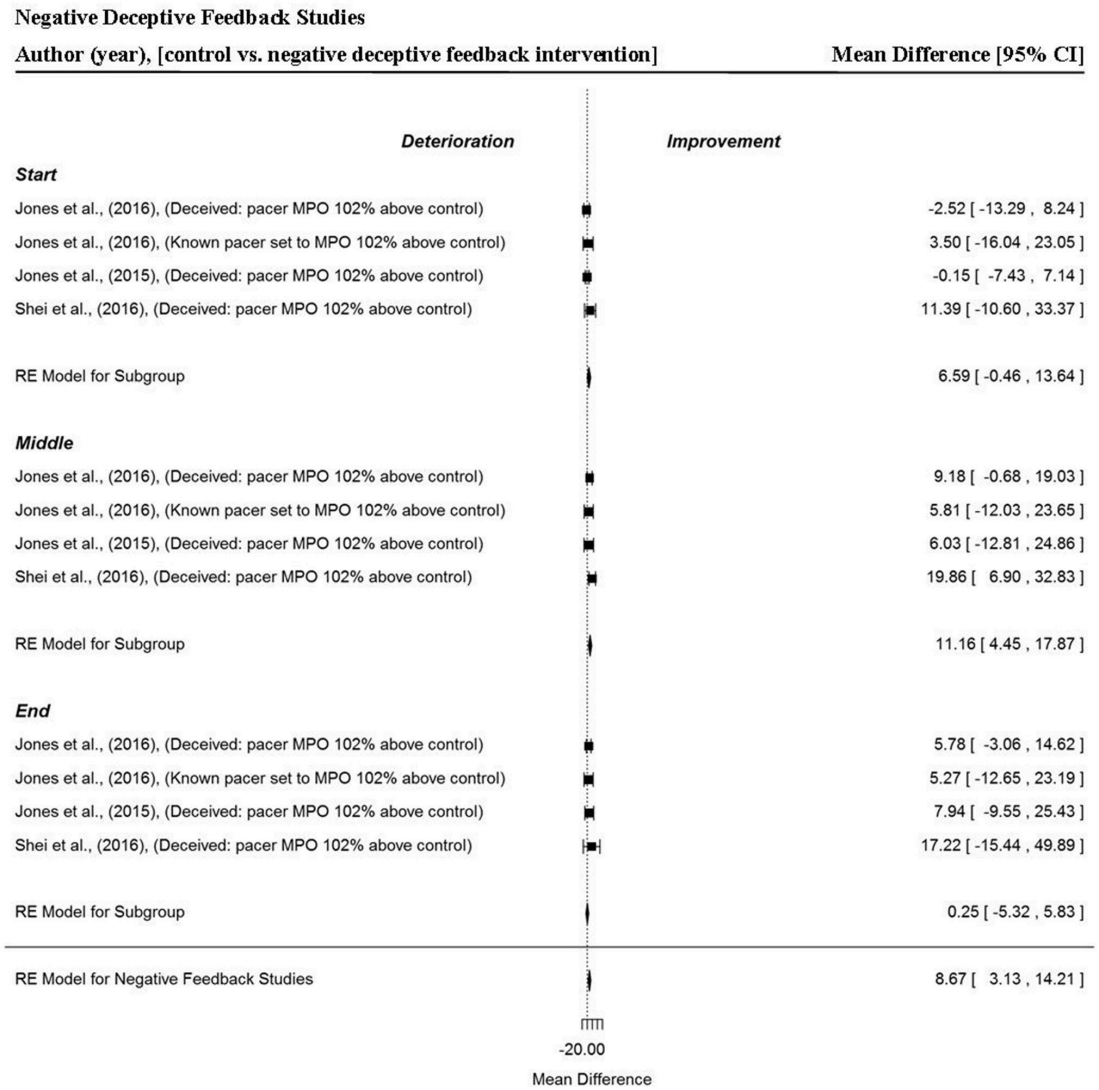
**Forest plot for negative deceptive feedback meta-analysis illustrating power output during start, middle and end sections compared to full feedback**. Squares represent individual study mean difference and the lines represent 95% CIs. The size of the square is proportional to the weight of the study within the meta-analysis. The diamond represents the overall mean difference for each split with the width of the diamond signifying the 95% CIs.

A total of four data points per trial section and overall trial MPO, were extracted from three studies that provided participants with negative deceptive feedback (Jones et al., 2016a,b; Shei et al., 2016). Generally, when participants were provided negative deceptive feedback there were small but significant improvements in power output (MD = 8.67 W, 95% CI = 3.13 to 14.21, *p* = 0.002) compared to receiving full and accurate feedback. In these studies, participants were racing against an avatar pacer they believed was programmed to mimic their previous trial MPO performance, however, in reality the pacer was programmed at a higher MPO (102%). Trial section data revealed a tendency for greater power outputs to be attained in the start section (MD = 6.59 W, 95% CI = −0.46 to 13.64, *p* = 0.067). A significant improvement in trial MPO was found in the middle section of trials (MD = 11.16 W, 95% CI = −0.46 to 13.64, *p* = 0.001). However, no significant changes were found in the end section of trials (MD = 0.25 W, 95% CI = −5.32 to 5.83, *p* = 0.929). Additionally, the width of the 95% CI were notably smaller compared to positive deceptive feedback, indicating there was less variability in overall performance and individual section MPO when participants were provided with negative deceptive feedback (Figure 8).

### Analysis of studies which investigated the manipulation of neutral feedback

**Figure 9:**
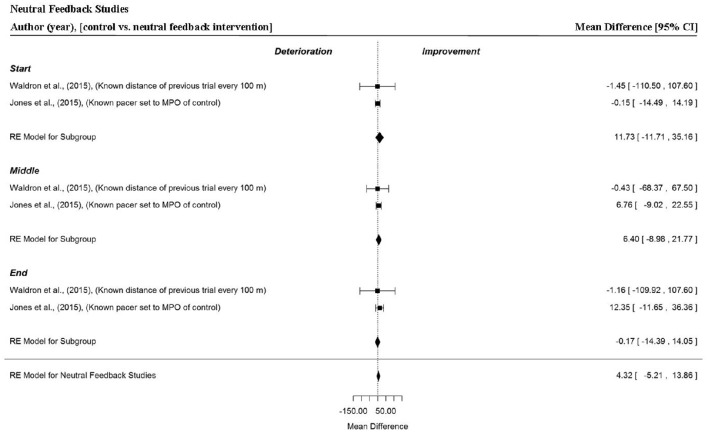
**Forest plot for neutral feedback meta-analysis illustrating power output during start, middle and end sections compared to full feedback**. Squares represent individual study mean difference and the lines represent 95% CIs. The size of the square is proportional to the weight of the study within the meta-analysis. The diamond represents the overall mean difference for each split with the width of the diamond signifying the 95% CIs.

Two data points per trial section MPO and for the whole trial MPO were extracted from two studies (Jones et al., 2016b; Waldron et al., 2015). Figure 9 shows that neutral feedback, where feedback was accurately given or where participants raced a virtual on-screen avatar that accurately represented the MPO of a previous performance, did not statistically influence overall MPO (MD = 4.32 W, 95% CI = −5.21 to 13.86, *p* = 0.374). There were no significant improvements in MPO across the start (MD = 11.73 W, 95% CI = −11.71 to 35.16, *p* = 0.327), middle (MD = 6.37 W, 95% CI = −8.98 to 21.77, *p* = 0.414) or end (MD = −0.17 W, 95% CI = −14.39 to 14.05, *p* = 0.981) sections.

### Analysis of studies which investigated the manipulation of no feedback

Data points were extracted from three different comparisons and three separate investigations (Swart et al., 2009; Williams et al., 2012; Smits et al., 2016). In these studies, where all visual and verbal performance feedback was withheld, no significant changes in power output were found (MD = 11.34 W, 95% CI = −12.67 to 35.34, *p* = 0.355). There were no significant changes in MPO in the start (MD = 7.23 W, 95% CI = −43.03 to 57.53, *p* = 0.778), middle (MD = 8.26 W, 95% CI = −25.31 to 41.84, *p* = 0.630) and end (MD = 20.94 W, 95% CI = −26.05 to 67.93, *p* = 0.382) sections of trials.

### Analysis of studies which investigated the manipulation of informed feedback

Three data points per trial section MPO and for the whole trial MPO were extracted from two studies (Jones et al., 2016a; Shei et al., 2016). Overall, a significant change in trial MPO was found when participants completed an informed trial following a negative feedback trial (MD = 10.55 W, 95% CI = 0.97 to 20.12, *p* = 0.031). In these studies, participants were either unaware (Jones et al., 2016a; Shei et al., 2016) or aware (Jones et al., 2016a) of the increased MPO of their previous trial (102%), represented by a virtual on-screen avatar. Participants completed a subsequent (informed) trial with the presence of a pacer and known MPO increase (102%) (Shei et al., 2016) or with no pacer (Jones et al., 2016a). Trial section data revealed a tendency for a small increase in power outputs in the end section of the trial (MD = 7.00 W, 95% CI = −1.01 to 15.00, *p* = 0.087). However, MPO across the start (MD = −2.01 W, 95% CI = −15.01 to 10.99, *p* = 0.762), and middle (MD = 9.01 W, 95% CI = −8.09 to 26.12, *p* = 0.302) sections of the trial were non-significant. Unexplained variance was low at the start (19%) and end (0%) of the trial, however substantially greater heterogeneity of variance (69%) was evident in the middle section of the trial.

### Analysis for heterogeneity

Unexplained variance, indicated by the *I*^2^ statistic, was low (0%) across all environmental and almost all feedback groups, indicating low heterogeneity for the start, middle and end sections respectively. An exception was the informed feedback group where a large percentage of unexplained variance was found for the middle section of trials. However, the meta-analysis included a small sample in the defined themes, with the exception of the heat-stress group, therefore the Q-test is likely underpowered for detecting true heterogeneity. Individual group results are outlined in Table 1.

## Discussion

Previously a number of reviews have been published concerning deception (Jones et al., 2013; Williams et al., 2014), decision making (Edwards and Polman, 2013; Renfree et al., 2014; Smits et al., 2014; McCormick et al., 2015) and neurophysiological determinants (Roelands et al., 2013) of pacing. However, to our knowledge this is the first meta-analysis to have quantified how the different environmental conditions and various forms of performance feedback used in studies to date have affected the pacing strategy exhibited during self-paced time-trials. By segmenting performance into three sections; the start, middle and end, this analysis provides further insight into how participants regulate their exercise under differing experimental conditions.

### Pacing and oxygen availability

The meta-analysis demonstrated in hypoxia trials participants' significantly reduced their MPO in comparison with their normoxia time-trials. However, the MPO for the start section of the hypoxia trials was not significantly different to the respective normoxia trials, which indicates there was a delay in the adjustment of the pacing strategy in hypoxia because it was only as the hypoxia trials progressed, that a significant reduction in MPO (in the middle and final sections) was observed in comparison with the normoxia trials. Notably in the hypoxia studies, participants began to inhale their allocated oxygen content for a period of time prior to the trials beginning (M ± SD: 16 ± 21 min) but were deceived concerning the nature of the inspired oxygen content. Therefore, despite inspiring gas mixtures with a reduced FiO_2_, well before the beginning of the hypoxia trials, participants began their hypoxic trials with a power output close to their “normal” normoxic trial pacing strategy. This similarity in starting MPO, irrespective of inspired FiO_2_, has been observed previously. It appears that when participants are blinded to the inspired oxygen content, there is a time lag between when the exercise begins and when the pacing strategy is changed compared to the normoxic condition. This suggests afferent feedback, from peripheral chemoreceptors in the aortic and carotid bodies sensing the F_i_O_2_ disturbance, takes a significant amount of time to be assimilated and acted upon. A time lag spanning between 30 and 60 s from the start of the exercise bout before a pacing adjustment is made has previously been reported (Peltonen et al., 2001a; Amann et al., 2006; Johnson et al., 2009; Henslin et al., 2013). Furthermore, it has been demonstrated that Phase I of the $\dot{V}$ O_2_ fast component is similar in hypoxic, normoxic, and hyperoxic conditions at the onset of exercise (Peltonen et al., 2001a), which may indicate there is an aspect of physiological pre-conditioning present at the start of a known exercise challenge, which occurs irrespective of current gaseous exchange conditions. However, once the exercise begins and afferent feedback is integrated then a decision is made which leads to a reduced central motor drive after a period of 30 s or more of the hypoxic trial. We must however acknowledge that this argument is speculative and might only hold for exercise challenges where there is a requirement for a high exercise intensity to be undertaken in reduced FiO_2_ conditions equivalent to low-moderate altitude, as was the case in this meta-analysis.

The reduction in MPO for the middle and end trial sections in hypoxic trials suggest participants adjusted their power output, as chemoreceptors detected the reduction in oxygen availability and peripheral capillary oxygen saturation, compared to the normoxic condition (Johnson et al., 2009). A further explanation for the reduction in MPO in the hypoxia trials is that due to a reduction in oxygen delivery the aerobic energy production was compromised, relative to the normoxic condition, and remained suppressed throughout the hypoxic trials (Peltonen et al., 2001a). This would potentially lead to a relatively greater anaerobic energy contribution in the hypoxic trials compared to the normoxic trial during middle and end sections of the trials. Subsequently, a greater proportion of the finite anaerobic capacity would be expended earlier in the hypoxia trial. This reduction in the rate and capacity for aerobic energy contribution coupled with the need for a greater utilization of the anaerobic capacity in the hypoxia trials, when integrated within brain centers, might have led to the motor cortex reducing central motor drive to the exercising skeletal musculature in order to reduce MPO and energy expenditure.

Notwithstanding the changes in the physiological milieu, there are also other factors to consider that would have potentially affected participants' MPO as they progressed through the hypoxic trials. It is plausible participants became increasingly aware of heightened sensory feedback informing the brain of the compromised metabolic rate during the hypoxic trials, and the subsequent development of fatigue and subsequently allocated time to deliberative decision-making. It has been hypothesized that incoming sensory afferent feedback, knowledge of the trial distance or duration (Swart et al., 2009), current momentary perceived effort influenced by prior experience (Mauger et al., 2009), current expectations (such as outcome and strategic goals) (Baden et al., 2004; Renfree et al., 2014) and knowledge of current physical capacities (Renfree et al., 2014) are integrated into decision making processes during self-paced exercise. At what point information resides in different brain centers and moves across sub-conscious, pre-conscious and conscious states is difficult to discriminate; however if the participant consciously perceives their actual level of exertion is above that which they would normally expect at a particular point in the trial then they might decide to reduce their level of effort accordingly (de Koning et al., 2011; Micklewright et al., 2015). It would seem likely that in the hypoxic trial participants consciously regulated their exercise intensity, as postulated by the psycho-biological model, and that a decision was made to reduce MPO in order to stave off developing fatigue apparent during the middle and end trial sections (Marcora, 2010).

It is worth highlighting however that one investigation reported no significant changes in MPO across all three trial sections during hypoxic trials compared to the normoxia trial (Clark et al., 2007). In this study when well-trained cyclists' first (5 min) time-trial was either at 3200- or 2200-m simulated altitude, they demonstrated a conservative approach in the start section of subsequent trials at lower simulated altitudes (200- and 1200-m respectively). The authors attributed this observation to an order effect rather than a learning effect, as participants had completed two familiarization trials in normoxia before completing trials at the various altitudes levels. Therefore, it seems apparent when participants are blinded to high simulated altitude they may subsequently select an inappropriate starting pace in subsequent trials at lower altitudes. Finally, it is worth noting that the pacing index change score for the trials in the Amann et al. (2006) study looks markedly different compared to the other studies (**Figure 12A**). In this study, a more aggressive pace was attempted in the hypoxia trial relative to the normoxia trial which led to a marked reduction in middle and end section MPO, suggesting that the initial pace was considerably misjudged.

The meta-analysis found no significant evidence that hyperoxia improves overall trial MPO or section MPO, despite previous literature supporting the benefits of inhaling hyperoxic air during time-trials (Peltonen et al., 1995, 1997, 2001a,b; Nielsen et al., 1999; Amann et al., 2006; Tucker et al., 2007). However, this might be attributable to the small sample size and so further research is warranted to determine the efficacy of inspiring hyperoxic gas mixtures and to understand how exercise is regulated when athletes inhale oxygen enriched air. It is important to mention that participants in the included studies were also blinded to the fact that they were inpsiring oxygen enriched air (Amann et al., 2006; Tucker et al., 2007). Therefore, whether knowledge of breathing hyperoxia encourages a conscious decision to upregulate exercise intensity or not, compared to a normoxic trial, requires further exploration.

### Pacing and heat-stress

When participants were exposed to hot and humid conditions the meta-analysis clearly demonstrated participant's MPO was significantly reduced compared to more temperate conditions. Notably there was less variability demonstrated in the RE model for the different sub-group sections (start, middle and end) in the heat-stress studies than in the studies which manipulated oxygen content. A possible explanation might be the nature of heat interventions, as a marked change in room temperature is easily identifiable whereas a change in the oxygen content of the air being inspired is not. It is therefore likely that appropriate adjustments to power output were made sooner in the heat trials due to earlier changes in afferent feedback.

In a similar trend to the hypoxia trials, the meta-analysis found power outputs produced during the start section of trials were similar to the control (temperate) trial. This finding is supportive of previous reports where the initial exercise intensity in hot conditions follows a similar pattern to temperate conditions for the first ~10 to 15 min of exercise, despite elevated skin temperatures, thermal sensation and perceived effort (Tatterson et al., 2000; Tucker et al., 2004; Ely et al., 2009; Périard et al., 2011). However, if participants are inexperienced at time-trialing in heat they can misjudge the start section of the trial by beginning the trial too aggressively (Schlader et al., 2011b; Castle et al., 2012). Notably, for a number of the heat-stress trials, the pacing index change score was relatively greater and lower for the start and end sections, respectively, compared to the temperate condition trial (Figure 12B). This indicates an inability for participants to adopt an optimal pacing strategy from the onset of the trial in the hot condition. Subsequently the pacing strategy was substantially adjusted as the trial progressed which might partly explain why a reduced performance outcome is observed compared to the temperate condition.

In some of the studies, the exposure to the heat-stress prior to the time-trial (i.e., during the standardized warm-up) was ≤ 15 min in duration (Castle et al., 2012; Schmit et al., 2016; VanHaitsma et al., 2016) or longer (Abbiss et al., 2008; Altareki et al., 2009). However, in the remaining studies either no mention was made as to participants being exposed to the heat-stress prior to the time-trials, or they did not expose their participants to heat-stress in the warm-up period (Tatterson et al., 2000; Tucker et al., 2004; Peiffer and Abbiss, 2011; Périard et al., 2011; Périard and Racinais, 2015, 2016). If we consider the role of afferent feedback, perceived effort and thermal sensation in exercise regulation during exercise in the heat, then the absence of any exposure to heat prior to the time-trial would delay the onset of the development of heightened afferent feedback from thermoreceptors and any subsequent increase in perceived effort and thermal sensation (Schmit et al., 2016). In this scenario, participants might begin with a starting pace at a higher level (similar to a temperate condition), than if they had been exposed to the heat-stress during the warm-up period, due to the role skin temperature has in mediating thermo-behavioral responses (Schlader et al., 2011a). If participants began the heat-stress trial too aggressively, due a lack of change in thermal sensation from no pre-time-trial heat exposure, then they would need to considerably adjust their exercise intensity in the middle section of the trial to avoid prematurely fatiguing. Unfortunately, due to time-trial duration varying markedly between studies in the meta-analysis, it was not possible to demonstrate if this trend was indeed apparent from a comparison of start, middle and end trial sections between those studies involving pre-time-trial heat exposure and those that did not.

Mean power output was observed to decline in the middle and end sections of hot condition trials compared to temperate trials across studies which investigated both short and long time-trial durations. An inspection of core temperature data from these studies revealed that during time-trials lasting ≤ 30 min and/or where participants weren't exposed to heat up to 20 min before the trial, core temperature changed in a similar fashion for trials in both temperate and hot conditions. This would suggest pacing was adjusted to avoid significant changes in core temperature (Nybo, 2008). The potential afferent driver of the adjustment in the pacing strategy is outside of the scope of this meta-analysis, however, it could be due to other factors such as significant increases in skin temperature (Jay and Kenny, 2009), perceived exertion (Crewe et al., 2008) and thermal sensation (Schlader et al., 2010).

It is well known that during exercise in hot conditions there is an increased skin blood flow, in order to dissipate heat, which compromises blood flow to the working musculature and in turn leads to a reduction in gross mechanical efficiency (Hettinga et al., 2007). This consequence appears to become more problematic in continuous exercise lasting over 30 min in duration, as the maintenance of blood pressure takes priority over blood flow to the skin and working musculature (Casa, 1999). As a result, differences between the rates of metabolic heat production and net heat loss, and the exchange of evaporative heat, will lead to increased core temperatures for longer duration time-trials in hot compared to temperate conditions (Jay and Kenny, 2009). This is due to the uncompensatable heat gain over the course of the trial, significantly impacting circulatory responses, leading to a decrease in $\dot{V}$ O_2_ and subsequent reduction in exercise intensity (Périard and Racinais, 2016).

Finally, some of the reduction of the power output in hot conditions could be attributable to discrepancies across studies which made no mention, or did not account for, one or more of the following factors: (i) whether the temperature of the control trial was in accordance with laboratory recommendations for exercise in a thermoneutral environment (18 to 23°C, relative humidity <70%) (Tucker et al., 2004; Altareki et al., 2009; Peiffer and Abbiss, 2011) and ii) if the relative increase in temperature from control to intervention was considered. For instance, the temperature difference between intervention and control conditions in the study by Altareki et al. (2009) was 22°C compared to 15°C by Périard et al. (2011). It is possible that the relative temperature increase from cool (≤18°C) to hot (≥30°C) conditions may be influential in terms of exercise regulation and may warrant further exploration. Third, there were a number of confounding variables in terms of comparing data across studies such as, a lack of information concerning the clothing participants wore during the trials, some studies not allowing participants to consume fluids during the trial (Altareki et al., 2009; Peiffer and Abbiss, 2011) and the lack of a consistent approach to using convective cooling methods providing equivalent volumes and rates of air flow to replicate the conditions that athletes encounter during outdoor competitions (25–40 km h^−1^ when cycling) (Dugas et al., 2009). Indeed, many of the heat intervention studies in the current meta-analysis provided less than the recommended volumes and rates of air flow during indoor cycling trials (M ± SD: 19.2 ± 8.9 km h^−1^). In addition, three studies did not mention whether convective cooling was used in heat trials (Castle et al., 2012; Schmit et al., 2016; VanHaitsma et al., 2016). It is, of course, also possible that researchers deliberately provided sub-optimal convective air flow to induce hyperthermia in their participants. Nonetheless, the differences in available air flow to dissipate heat may partly explain the variation of power output between individual studies.

### Pacing and pre-cooling interventions

In general, pre-cooling interventions aim to counter the effects of exercising in heat by cooling the body prior to the exercise to increase the “thermal reservoir” (Nielsen, 1994; Levels et al., 2012). In the current meta-analysis the pooled data for studies investigating pre-cooling interventions demonstrated no significant effect on overall trial or trial section MPO compared to the control trial, despite a number of the studies having reported small increases in MPO following 20 min of cold water (14°C) lower body immersion (Duffield et al., 2010), ingestion 900 mL of cold fluid (2°C) (Byrne et al., 2011) or after wearing a cooling vest (18 min during warm-up) (Gonzales et al., 2014). Some of the included studies in the meta-analysis had reported lowered skin and muscle temperatures, and perceived thermal sensation for their participants following the pre-cooling intervention prior to their time-trials. These parameters have been suggested to reduce thermoregulatory responses by delaying the redistribution of cardiac output to the periphery and sweat responses for heat dissipation (Casa, 1999), during the early stages of exercise (~15 min) following pre-cooling strategies, although once exercise exceeds ~10 min, any physiological and perceptual changes induced by pre-cooling appears to dissipate (Minett et al., 2011). Therefore, any change in the pacing strategy resulting from pre-cooling interventions would be likely detected in the start section of trials. In the current meta-analysis, the exercise duration of the included studies were considerably longer than 10 min (M ± SD: 40 ± 22 min), and so the start section took a number of minutes to complete however no significant changes in MPO for the start section of the trials was detected. A closer inspection of core temperature data from the original manuscripts revealed that with the exception of ingesting 900 mL of cold fluid (Byrne et al., 2011), the pre-cooling strategies of the included studies were unsuccessful in lowering body temperature before the start of the time-trial, compared to the control condition (Duffield et al., 2010; Barwood et al., 2012; Gonzales et al., 2014). This is likely attributable to the effectiveness of the intervention itself and the time period between the completion of the pre-cooling method and warm-up or between the warm-up and the start of exercise (M ± SD: 12 ± 8 min).

Given the findings of the meta-analysis, careful consideration should be given as to whether pre-cooling interventions are of benefit to athletes' performances when competing in a hot environment, especially as the logistical aspects of undertaking pre-cooling interventions can be disruptive. In addition, if the pre-cooling intervention has the potential to lower muscle temperature then it might actually be detrimental for performance in short duration (<12 min) events (Levels et al., 2012). It is worth noting however that with only four studies meeting the inclusion criteria for this meta-analysis, our findings are likely to be underpowered and further research in this area is needed to fully elucidate the effects of pre-cooling interventions, particularly in terms of exercise regulation during the start section of the trials.

Finally two studies (Duffield et al., 2010; Byrne et al., 2011) had cyclists refrain from consuming water during their respective trials. Previous reports have shown that removing the physical act of drinking may alter the participant's perception of thirst, and therefore, potentially influence their motivation and pacing to limit further fluid loss (Dugas et al., 2009; Cheung et al., 2015). This was demonstrated by Dugas et al. (2009) who restricted the fluid consumption of participants below *ad libitum* and found time-trial performance was impaired in male cyclists. It's plausible that the effectiveness of the pre-cooling methods included in the analysis, may have been confounded by the absence of fluid consumption in some studies.

### Pacing and feedback manipulations

**Figure 10:**
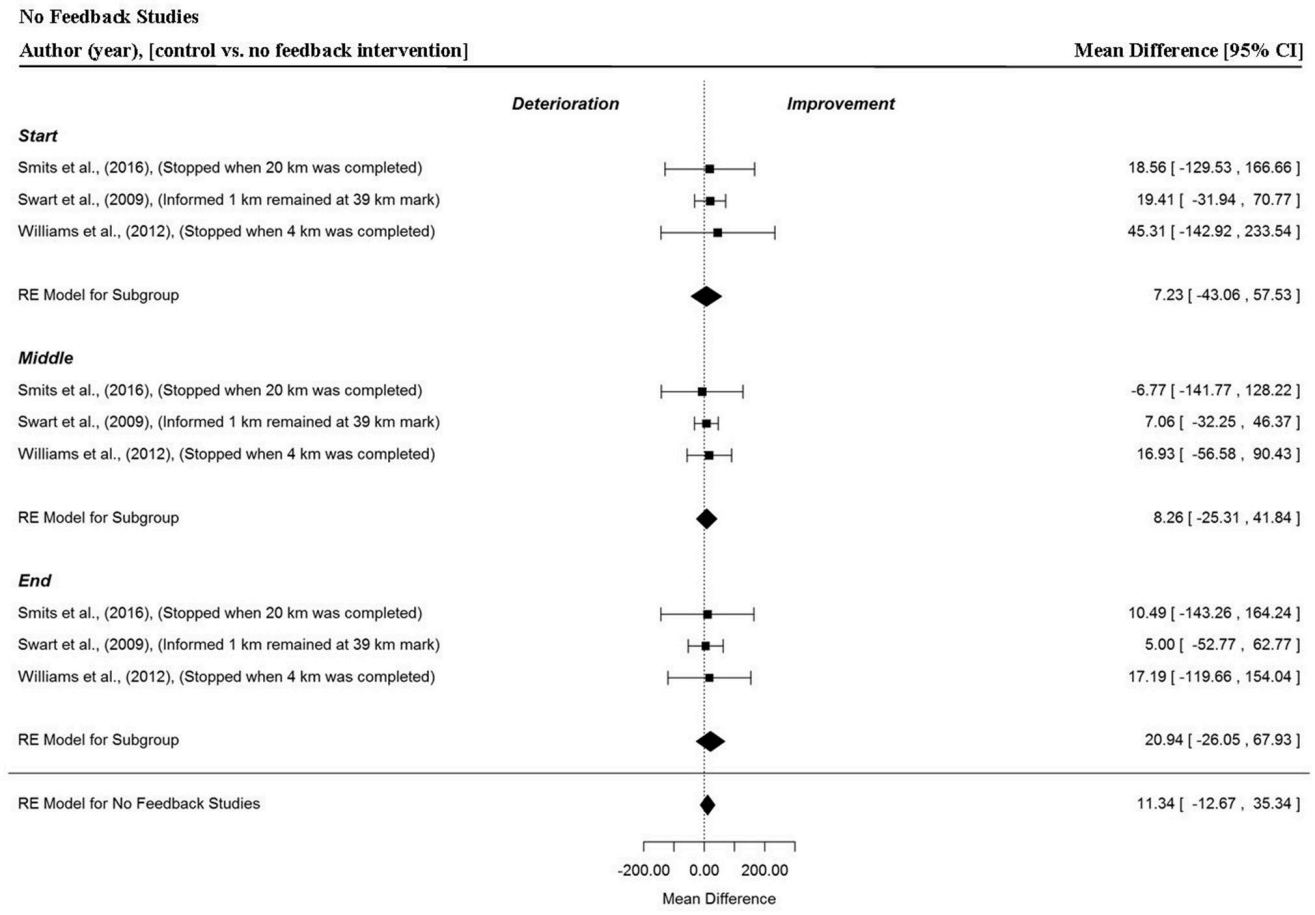
**Forest plot for no feedback meta-analysis illustrating power output during start, middle and end sections compared to full feedback**. Squares represent individual study mean difference and the lines represent 95% CIs. The size of the square is proportional to the weight of the study within the meta-analysis. The diamond represents the overall mean difference for each split with the width of the diamond signifying the 95% CIs.

**Figure 11:**
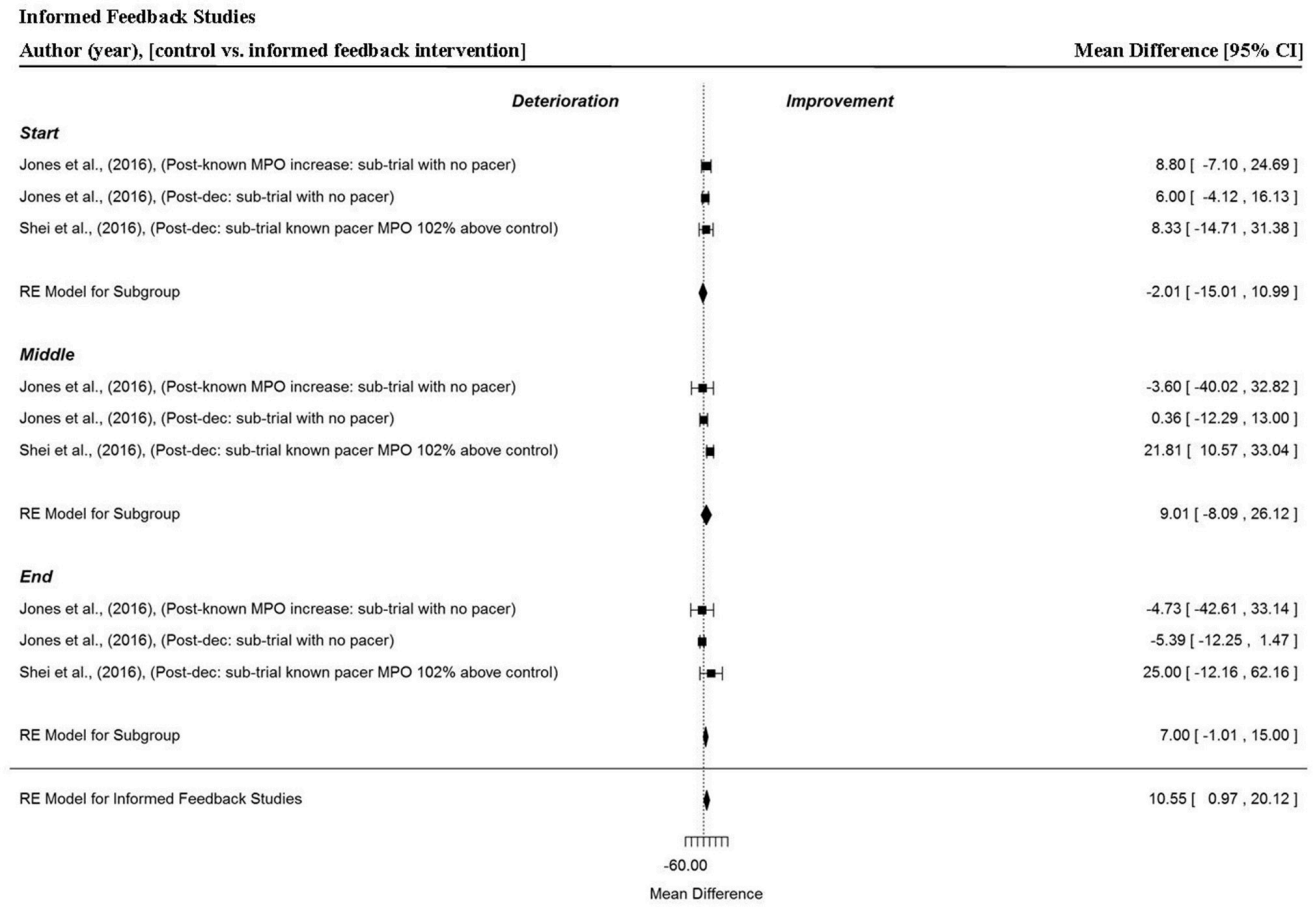
**Forest plot for informed feedback meta-analysis illustrating power output during start, middle and end sections compared to full feedback**. Squares represent individual study mean difference and the lines represent 95% CIs. The size of the square is proportional to the weight of the study within the meta-analysis. The diamond represents the overall mean difference for each split with the width of the diamond signifying the 95% CIs.

Overall, positive, neutral and no feedback had no significant effect on overall or individual segment MPO (Figures 7, 9, and 10, respectively). However, negative feedback, or more specifically performance deception feedback, did elicit significant improvements in performance and individual segment power output. In these studies, participants were racing against a virtual on-screen pacer they believed was programmed to mimic MPO from their previous best trial performance, however in reality the pacer was programmed with a MPO which was 2% greater (Jones et al., 2016a,b; Shei et al., 2016). Participants tended to produce an increased power output at the start of the “deception” trial and also produced a significantly greater power output during the middle section. The variability of power output was relatively low in these trials which might suggest the change in power output, caused by the deception, was sufficient to improve performance but not so great as to be intolerable. Previous work by Stone et al. (2012) found when participants were deceived of a 2% increase in the MPO they demonstrated a greater anaerobic energy contribution at 90% of a 4000-m time-trial and a concomitant significant improvement in power output. The meta-analysis did not find an improvement for the end section of trials, however this might be because trials were segmented into thirds rather than 10% bins as in the Stone et al. (2012) study. The meta-analysis also demonstrated informed feedback (Figure 11; where participants completed a time-trial subsequent to being informed the MPO of a pacer in their previous trial was set to a greater exercise intensity than their baseline performance) led to improved trial performance compared to an original (baseline) time-trial.

**Figure 12:**
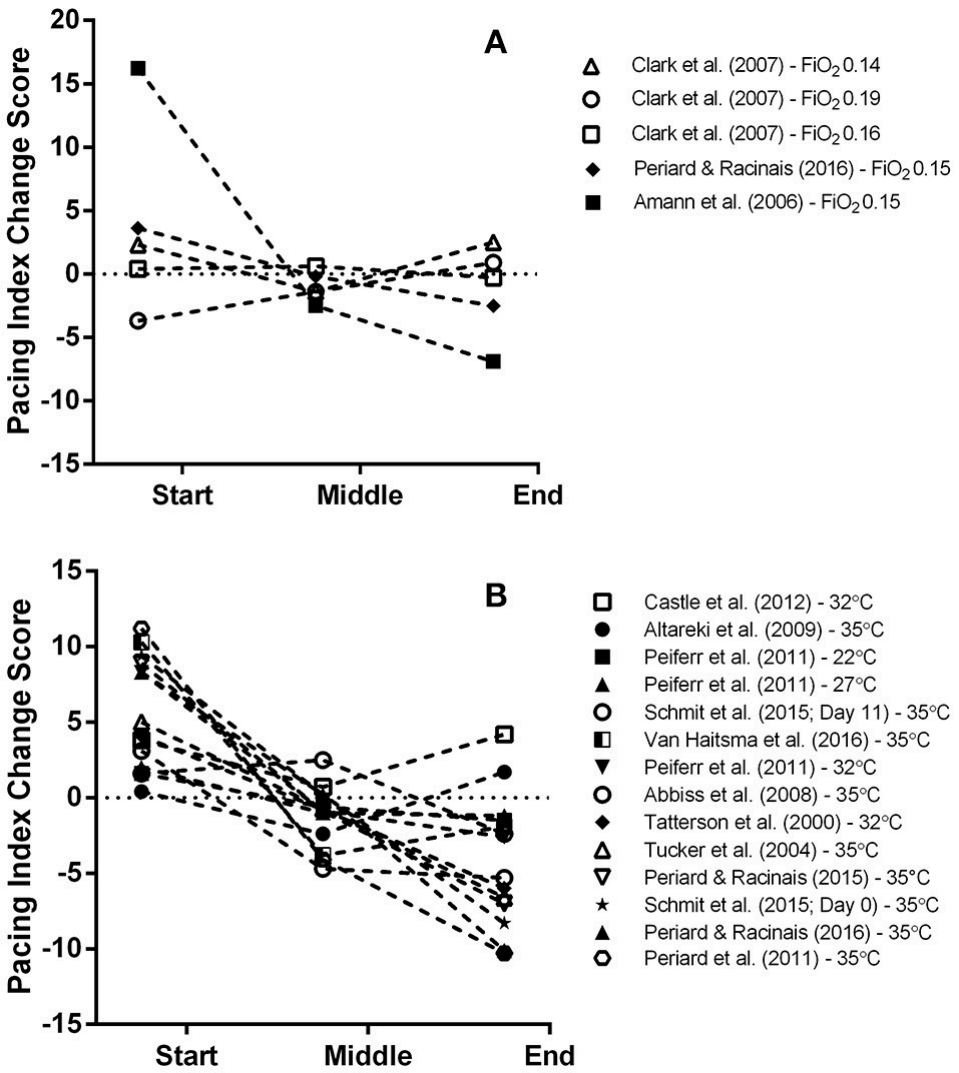
**Exploratory graphical analysis of the pacing index change score difference between control and hypoxic condition (A)** and control and heat-stress condition **(B)** for each individual segment. For an explanation of how the pacing index change score was calculated please refer to section Data analysis of the Methods.

Collectively, these findings from different forms of feedback indicate that participants are preconditioned to start exercise at a similar intensity in the early stages of a trial despite the absence or manipulation of feedback. However, negative feedback, such as deceiving participants about their actual power output can achieve subtle increases in power output during the start and middle trial sections and an improved overall trial MPO. However, recent research suggests the presence of an on-screen avatar pacer in these studies, provides the additional motivation of a “competitor,” and may partly explain the increases in power output observed (Stone et al., 2012; Shei et al., 2016). It is worth noting however, that the neutral feedback condition in the meta-analysis did not demonstrate a significant improvement in trial MPO when a “competitor” pacer was present, as compared with the baseline (control) time-trial where no pacer was present.

## Limitations of the meta-analysis

A limitation of the meta-analysis was that a large number of studies were discounted because they did not report power output data. Power output was decided upon as the performance metric of choice for the analysis as measurement error data is consistently reported for this parameter in the pertinent literature. Consequently, a number of pacing studies providing velocity or time data were excluded, despite meeting all other criteria. Additionally, three studies in rowing, where power output was measured, were excluded because once studies were allocated into the defined themes the sample size in each group was not sufficient to be included in a meta-analysis (Mujika et al., 2010; Taylor et al., 2014; Murray et al., 2016). As a consequence of these constraints, modes of exercise researched in the literature such as running, swimming, skating and rowing were excluded. The exclusion of a large quantity of studies from the pacing literature subsequently impacted the sample size, particularly when studies where allocated into themes for the meta-analysis. For example, the null effect found with regard to pre-cooling interventions in hot conditions were certainly impacted by the small sample size (*n* = 4) and the diverse range of methods and experimental designs. Interpretations of exercise regulation in the included themes should therefore be viewed with caution, until additional studies suitable for a meta-analysis, are available to provide greater understanding of exercise regulation within the pacing literature. A further limitation of the analysis is that the pacing resolution was compromised, because trial power output data could only be segmented in to three sections (start, middle and end sections) rather than for example, 10% bins which would have provided greater insight into changes within the pacing strategy for the various interventions. This was due to the large variability in trial duration and distance being used in the included studies. Finally, with the exception of one study that recruited one female participant into a male cohort (Périard and Racinais, 2015), only male participants' were recruited in the included studies. Therefore, the findings have an obvious gender bias, as unfortunately, there is a dearth of studies to date investigating changes in the pacing strategies exhibited by females.

## Application and future recommendations

We recommend that researchers be attentive to the criteria for developing high quality research, such as those outlined in the PEDro scale (Machado et al., 2016) to provide more robust datasets to undertake a meta-analysis. Additionally, it would be helpful if research investigating different manipulations on pacing provided a pacing index, which is a relatively simple measure to report, rather than focusing primarily on overall performance outcomes. The pacing index allows for direct comparisons between genders, age or in the instance of a meta-analysis, between different studies of similar themes (Le Meur et al., 2011; Wu et al., 2014).

The notable delay in exercise regulation during the start section of time-trials in hypoxia and heat-stress highlights the importance of taking into account the exposure time to the environmental stressor prior to and during a time-trial or a competitive race. Experimental investigators and coaches need to be mindful of the effects that exposure to different environmental stressors will have on the participant's/athlete's pacing strategy and overall performance. From a practical perspective, coaches and athletes need to consider adapting the “normal” race pacing strategy to the environmental conditions to mitigate against an error in pace judgment occurring from the start of the race. For athletes who compete globally, their race preparation needs to include practicing different pacing strategies under different race conditions to arrive at the optimal pacing strategy for each environmental condition. Coaches might consider the use of *subtle* deceptive negative feedback in training to elicit a change in pacing strategy and athlete performance. Having achieved a performance improvement there appears to be some evidence that the performance can be maintained even after the deception is revealed, however in a real-world setting this practice would be controversial, as there are ethical, integrity and moral aspects to be considered, which could be potentially harmful to the athlete and to the relationship with the coach.

Finally, there are a number of specific considerations for experimental investigators that were highlighted in conducting this meta-analysis. Firstly, when undertaking heat-stress trials in laboratory settings it is important consider: (i) the amount of change in temperature between control and intervention trials, (ii) whether the control trial represents conditions appropriate for a thermo-neutral trial, (iii) the availability of fluids for *ad libitum* drinking, (iv) the clothing worn and (v) adopting convective cooling methods that reflect outdoor conditions. Research studies manipulating the oxygen content of inspired air should be mindful that order effects may occur when trials simulating moderate altitude are followed by lower altitude trials. Studies manipulating feedback using a pacer/pacing device should consider the size of the effect this might have on the primary outcome measures exhibited by the participants relative to the size of the effect from the form of feedback being manipulated.

## Conclusions

The meta-analysis demonstrated that in trials where the environmental conditions were manipulated (e.g., hypoxia, hyperoxia or heat-stress) MPO was generally not significantly different to the control (normoxic or temperate) condition for the start section. However, MPO in the middle and end sections was found to be significantly reduced when participants were exposed to different levels of hypoxia and heat-stress. The available data demonstrated hyperoxia, pre-cooling strategies and some forms of feedback (positive, neutral and no feedback) did not significantly change trial or section MPO compared to their control condition. However, negative feedback, such as deceiving participants about their actual power output when competing against a virtual competitor, can result in small but significant increases in power output during the start and middle sections of trials and an improved trial MPO. Once informed of the deception, the meta-analysis also demonstrated participants can still produce an improved MPO in comparison to their original baseline time-trial.

## Author contributions

MD, BC, MW, SS, LG, PS, KT: (1) substantial contributions to the conception and design of research, acquisition of literature, analysis and interpretation of data for the manuscript; (2) substantial contributions drafting the work or revising it critically for important intellectual content; (3) substantial contributions to the final approval of the version to be published; (4) all authors acknowledge and agree to be accountable for all aspects of the work in ensuring that questions related to the accuracy or integrity of any part of the work are appropriately investigated and resolved.

### Conflict of interest statement

The authors declare that the research was conducted in the absence of any commercial or financial relationships that could be construed as a potential conflict of interest.
